# Histone Deacetylase Inhibitors Target DNA Replication Regulators and Replication Stress in Ewing Sarcoma Cells

**DOI:** 10.1158/2767-9764.CRC-25-0058

**Published:** 2025-06-27

**Authors:** Stacia L. Koppenhafer, Elizabeth L. Geary, Mary V. Thomas, Emma E. Croushore, Jessica A.O. Zimmerman, Jenna M. Gedminas, Dawn E. Quelle, Rebecca D. Dodd, David J. Gordon

**Affiliations:** 1 Division of Pediatric Hematology/Oncology, Department of Pediatrics, University of Iowa, Iowa City, Iowa.; 2 Department of Neuroscience and Pharmacology, Carver College of Medicine, University of Iowa, Iowa City, Iowa.; 3 Department of Internal Medicine, Carver College of Medicine, University of Iowa, Iowa City, Iowa.

## Abstract

Histone deacetylases (HDAC) regulate diverse pathways in cancer cells. Previously, we identified that Ewing sarcoma tumors, which are caused by a translocation between the *EWSR1* and *FLI1* genes (EWS::FLI1), are sensitive to drugs that target DNA replication, including the RRM1 and RRM2 subunits of ribonucleotide reductase, and the ATR–checkpoint kinase 1 (CHK1)–WEE1 signaling pathway. In this study, we identified that multiple HDAC inhibitors, including fimepinostat, romidepsin, and panobinostat, downregulate the levels of the RRM1, RRM2, CHK1, and WEE1 proteins in Ewing sarcoma cells and impair DNA replication. Moreover, transcriptome analyses identified that HDAC inhibitors downregulate the expression of multiple components of the prereplication complex, including the minichromosome maintenance complex 2–7 (MCM2–7) proteins and CDT1, that are essential for genomic DNA replication. Additionally, proteomic studies identified that HDAC inhibitors also downregulate the level of the BRD4 protein, a BET bromodomain protein that regulates both the transcriptional program of the EWS::FLI1 oncoprotein and DNA replication. Overall, these results provide novel insight into the molecular mechanisms by which HDAC inhibitors target cancer cells, regulate DNA replication, and inhibit the cellular response to DNA replication stress.

**Significance:**

In this work, we identify that HDAC inhibitors broadly disrupt DNA replication and the response to replication stress in Ewing sarcoma cells by downregulating RRM1, RRM2, CHK1, and WEE1. Notably, HDAC inhibitors also reduce MCM2–7 proteins, which are essential for prereplication complex helicase function in replication initiation and elongation, and CDT1, which loads MCM2–7 onto DNA.

## Introduction

The standard treatment for Ewing sarcoma—cytotoxic chemotherapy combined with surgery and/or radiation—has remained largely unchanged over the past three decades, yielding suboptimal outcomes and significant treatment-associated morbidities, such as renal toxicity, heart failure, and secondary malignancies (1, 2). This highlights the urgent need to identify tumor-specific vulnerabilities in Ewing sarcoma that can improve therapeutic outcomes and minimize toxicity.

DNA replication stress is a hallmark of cancer cells and a potential therapeutic target (3–7). In Ewing sarcoma tumors, DNA replication stress is driven by various factors, including cell-cycle dysregulation, increased frequency of R-loops, elevated levels of the helicase SLFN11, loss of the cohesin subunit STAG2, upregulation of nucleoside transporters, haploinsufficiency of the *EWSR1* gene, functional loss of ATM activity, and a BRCA1-deficient phenotype (refs. 8–16; bioRxiv 2024:2023.04.30.538578). Furthermore, Ewing sarcoma cells are particularly sensitive to further exacerbation of DNA replication stress through inhibition of ribonucleotide reductase (RNR), which is the rate-limiting enzyme in the synthesis of dNTPs, and blockade of the replication stress response using ATR, checkpoint kinase 1 (CHK1), and WEE1 inhibitors (3, 17–29). Clinical trials are currently evaluating combinations of these inhibitors with standard chemotherapy.

In addition to the ATR–CHK1–WEE1 axis, cells employ a preemptive strategy to mitigate replication stress by licensing dormant DNA replication origins during late M-phase and G_1_ phase. These dormant origins serve as backups to ensure DNA replication completion if replication forks stall or collapse (30–32). The minichromosome maintenance complex 2–7 (MCM2–7) forms the ATPase core of the prereplication complex (pre-RC) helicase that licenses DNA replication origins. The MCM2–7 proteins are highly abundant in proliferating cells, and by the end of the G_1_ phase, the amount of DNA-bound MCM2–7 exceeds the number of active origins used during a normal S-phase (33, 34). Importantly, partial reductions in MCM2–7 levels sensitize cells to DNA replication stress without impairing normal replication (35–39).

We previously demonstrated that histone deacetylase (HDAC) inhibitors downregulate the expression of the RRM1 subunit of RNR (40). In this study, we identify that HDAC inhibitors more broadly disrupt DNA replication and the cellular response to DNA replication stress by downregulating the expression of RRM1, RRM2, CHK1, and WEE1 in Ewing sarcoma cells. Notably, we also found that HDAC inhibitors reduce the levels of the MCM2–7 proteins and the CDT1 protein, which loads the MCM2–7 complex onto DNA. Finally, recognizing that HDAC inhibitors target multiple pathways beyond DNA replication, we demonstrate that BRD4, a transcriptional cofactor for the EWS::FLI1 oncoprotein and a regulator of DNA replication, and survivin (BIRC5), an antiapoptotic protein, are also targets of HDAC inhibitors in Ewing sarcoma cells (41–45). Overall, these results provide novel insight into the mechanism of how histone acetylation regulates DNA replication in Ewing sarcoma tumors.

## Materials and Methods

### Cell lines and culture

The TC71 (RRID:CVCL_2213) and EW8 (RRID:CVCL_1658) cell lines were provided by Dr. Kimberly Stegmaier (Dana-Farber Cancer Institute, Boston, MA), the AGPN (RRID:CVCL_X981) cell line was obtained from the Childhood Cancer Repository (Children’s Oncology Group), and the HT1080 (RRID:CVCL_0317), HEK-293T (RRID:CVCL_1926), and U2OS (RRID:CVCL_0042) cell lines were obtained from ATCC. The RD (RRID:CVCL_1649) cell line was provided by Dr. Munir Tanas (University of Iowa, Iowa City, IA), and the ES6 (RRID:CVCL_1202) cell line was obtained from the Childhood Solid Tumor Network at St. Jude Children’s Research Hospital. The cell lines were maintained at 37°C in a 5% CO_2_ atmosphere and grown in DMEM supplemented with 10% FBS, 100 IU mL^−1^ penicillin, and 100 μg mL^−1^ streptomycin. DNA fingerprinting confirmation of cell lines was performed using the short tandem repeat method, and cell lines were used within 8 to 10 passages of thawing.

### Chemical compounds

Panobinostat (HY-10224), romidepsin (HY-15149), and YM155 (HY-101-94) were obtained from MedChemExpress. Doxycycline (J67043-AE) and puromycin (J67236.8EQ) were obtained from Thermo Fisher Scientific.

### Cell viability assay

Cell proliferation was measured using the alamarBlue (resazurin; Sigma-Aldrich, #R7017) fluorescence assay, as previously described (46, 47). Approximately 5 × 10^4^ cells were plated per well of a 96-well plate, after which the cells were exposed to a range of drug concentrations for 72 hours. Fluorescence readings were then obtained after adding alamarBlue using a FLUOstar Omega microplate reader (BMG LABTECH). IC_50_ values were calculated using log-transformed and normalized data (GraphPad Prism 10.1.0; RRID:SCR_002798).

### Protein isolation and immunoblotting

Protein extracts for immunoblotting were prepared by incubating cells in RIPA buffer (Boston BioProducts) that was supplemented with protease and phosphatase inhibitors (Halt Protease and Phosphatase Inhibitor Cocktail, EDTA-free; Thermo Fisher Scientific) for 20 minutes. Supernatants were collected following centrifugation, and SDS-PAGE was used to separate proteins, which were then transferred to polyvinylidene difluoride membranes (Millipore). Membranes were cut and divided, using a prestained protein ladder (Thermo Fisher Scientific) for molecular weight landmarks, prior to hybridization with antibodies. Chromatin-bound proteins were isolated using a Subcellular Protein Fractionation Kit for Cultured Cells (Thermo Fisher Scientific) according to the manufacturer’s instructions. Antibodies to the following proteins were used in the immunoblots: FLI1 (Abcam, #ab133485, 1:1,000, RRID:AB_2722650), actin (Cell Signaling Technology, #4970, 1:5,000; RRID:AB_2223172), α-tubulin (Proteintech, # 66031-1-Ig, 1:5,000, RRID:AB_11042766), histone H3 (Cell Signaling Technology, #4499, 1:1,000, RRID:AB_10544537), acetyl-histone H3-Lys 9 (Cell Signaling Technology, #9649, 1:1,000, RRID:AB_823528), acetyl-histone H3-Lys 14 (Cell Signaling Technology, #7627, 1:1,000, RRID:AB_10839410), acetyl-histone H3-Lys 18 (Cell Signaling Technology, #13998, 1:1,000, RRID:AB_2783723), acetyl-histone H3-Lys 27 (Cell Signaling Technology, #8173, 1:1,000, RRID:AB_10949503), acetyl-histone H3-Lys 56 (Cell Signaling Technology, #4243, 1:1,000, RRID:AB_10548193), c-myc (Cell Signaling Technology, #18583, 1:1,000, RRID:AB_2895543), BRD4 (Cell Signaling Technology, #13440, 1:1,000, RRID:AB_2687578), replication protein A (RPA2; Bethyl Laboratories, A300-244A, 1:2,000, RRID:AB_185548), MCM2 (Cell Signaling Technology, #3619, 1:1,000, RRID:AB_2142137), MCM3 (Proteintech, #15597-1-AP, 1:500, RRID:AB_2141973), MCM5 (Proteintech, #11703-1-AP, 1:1,000, RRID:AB_2235162), CDT1 (Cell Signaling Technology, #8064, 1:1,000, RRID:AB_10896851), RRM1 (Cell Signaling Technology, #8637, 1:1,000, RRID:AB_10896851), RRM2 (Proteintech, #11661-1-AP, 1:500, RRID:AB_2180392), CHK1 (Cell Signaling Technology, #2360, 1:1,000, RRID:AB_11217623), WEE1 (Cell Signaling Technology, #13084, 1:1,000, RRID:AB_2713924), and survivin (Cell Signaling Technology, #2808, 1:1,000, RRID:AB_2063948). Immunoblots were analyzed and quantified using Fiji (RRID:SCR_002285).

### siRNA transfection

Cells (1.5–3 × 10^5^) were plated 1 day prior to transfection in six-well plates. Cells were transfected with siRNA using Lipofectamine RNAiMax (Thermo Fisher Scientific) according to the manufacturer’s instructions. siControl was obtained from Cell Signaling Technology (#6568; refs. 48, 49). siMCM2 was obtained from IDT (hs.Ri.MCM2.13.1 and hs.Ri.MCM2.13.2). siHDAC1, siHDAC2, siHDAC3, and siHDAC8 were all obtained from Dharmacon (ON-TARGETplus).

### RNA sequencing and analysis

RNeasy Plus Mini Kit (QIAGEN) was used to isolate RNA from cell lines. Samples were submitted to the Iowa Institute of Human Genetics Core Facility for sequencing. Samples were barcoded, pooled, and sequenced on an Illumina NovaSeq 6000 (Illumina) to obtain a minimum of 30 million paired-end 100 bp reads per sample. FastQC was used to assess the quality of the sequencing reads. Reads were then mapped against the human reference genome (hg38) using the STAR aligner (STAR, RRID:SCR_004463) and summarized at the exon level using featureCounts (RRID:SCR_012919). Raw counts were transformed using the cpm function in edgeR for sample clustering and principal component analysis. No outlier samples were identified or removed from the analysis. The DESeq2 package (DESeq, RRID:SCR_000154) was used for the identification of differentially expressed genes. Differentially expressed gene data were analyzed for gene set enrichment and transcription factor enrichment analysis using ShinyGO 0.77 (RRID:SCR_019213), Enrichr (RRID:SCR_001575), and iDEP.96 (50–52). The sequencing data are available on the Gene Expression Omnibus under the accession number GSE280573. Previously published RNA sequencing (RNA-seq) data for Ewing sarcoma cell lines treated with siRNA targeting EWS::FLI1 are available on the Gene Expression Omnibus under the accession number GSE263504 (53). Previously published RNA-seq data for Ewing sarcoma cell lines with CRISPR-mediated knockout of RRM1 are available on the Gene Expression Omnibus under the accession number GSE215881.

### qRT-PCR

Total RNA was extracted from cells using an RNeasy kit (QIAGEN) following the manufacturer’s instructions. One microgram of total RNA was reverse-transcribed into first-strand cDNA using random hexamer primers and SuperScript III Reverse Transcriptase (Thermo Fisher Scientific). qRT-PCR was performed on the ViiA 7 Real-Time PCR System (Life Technologies) using SYBR Select Master Mix (Thermo Fisher Scientific). The qRT-PCR primers for BRD4, EWS::FLI1, and GAPDH were 5′-GAG​CTA​CCC​ACA​GAA​GAA​ACC-3′, 5′-TCC​TAC​AGC​CAA​GCT​CCA​AGT​C-3′, and 5′-CTG​GGC​TAC​ACT​GAG​CAC​C-3′, respectively. Reactions were performed in triplicate, and gene expression was normalized to GAPDH.

### EdU labeling and detection

Detection of DNA replication was performed in triplicate using a Click-iT 5-ethynyl-2′-deoxyuridine (EdU)-488 kit (Thermo Fisher Scientific; ref. 17). Briefly, cells were labeled with 10 mmol/L EdU for 1 hour and then harvested using trypsin and fixed using the Click-iT fixative buffer. Cells were then washed by centrifugation and resuspended in Click-iT saponin-based permeabilization and wash reagent. Click-iT Plus reaction cocktail was added to the cell suspension for 30 minutes, after which the cells were washed by centrifugation. Flow cytometry was performed on a BD LSR II flow cytometer.

### Doxycycline-inducible shRPA2

shERWOOD UltramiR lentiviral-inducible short hairpin RNA (shRNA) plasmids targeting shRPA2 were obtained from transOMIC Technologies. Lentivirus was produced by transfecting HEK-293T cells with the shRNA plasmid and packaging plasmids (psPAX2 and pMD2.G) according to the FuGENE 6 (Roche) protocol. For the lentiviral transduction, Ewing sarcoma cells were incubated with 2 mL of virus and 6 mg/mL of polybrene (Sigma-Aldrich) for 12 to 16 hours. Cells were selected in 1 μg/mL puromycin 48 hours after transduction.

### RPPA

Reverse phase protein array (RPPA) analysis of cell lines was performed by the RPPA Core Facility at the MD Anderson Cancer Center. Cells were provided to the core facility as frozen pellets, and the protein extraction, data normalization, and analysis were performed according to facility protocols (https://www.mdanderson.org/research/research-resources/core-facilities/functional-proteomics-rppa-core/education-and-references.html; RRID:SCR_016649).

### Cancer Dependency Map

Gene expression and CRISPR dependency data from the Cancer Dependency Map resource were accessed through the DepMap Portal (depmap.org; RRID:SCR_017655; refs. 54, 55).

### Survival analysis

Kaplan–Meier curves were analyzed for significance using the log-rank (Mantel–Cox) test with the R2 Genomics Platform (https://r2.amc.nl; RRID:SCR_025770) and the Ewing Sarcoma (Savola) dataset (56).

### Statistical analysis

Statistical analyses were conducted using GraphPad Prism 10.1.0 (RRID: SCR_002798). Single comparisons were analyzed by the unpaired two-tailed *t* test. Multiple comparisons were analyzed by one-way ANOVA with the Tukey *post hoc* multiple comparisons test. *P* values of less than or equal to 0.05 were considered significant.

### Data availability

Raw data are available in the Supplementary Supporting Data Values file. Unedited immunoblots are available in the unedited blot and gel images file. The sequencing data are available on the Gene Expression Omnibus under the accession number GSE280573. Previously published RNA-seq data for Ewing sarcoma cell lines treated with siRNA targeting EWS::FLI1 are available on the Gene Expression Omnibus under the accession number GSE263504 (53). Previously published RNA-seq data for Ewing sarcoma cell lines with CRISPR-mediated knockout of RRM1 are available on the Gene Expression Omnibus under the accession number GSE215881.

## Results

### HDAC inhibitors decrease RRM1, RRM2, CHK1, and WEE1 protein levels

Ewing sarcoma cells are sensitive to the dual inhibition of RNR and CHK1, a key mediator of the response to DNA replication stress (17, 19–22). Previously, we identified that HDAC inhibitors downregulate the RNR subunit RRM1 protein expression in Ewing sarcoma cells (40). Notably, fimepinostat, a dual inhibitor of HDAC and PI3K, was recently shown in leukemia cell lines to reduce the levels of additional proteins with critical roles in DNA replication, including RRM2, CHK1, and WEE1 (57, 58). Consequently, we began this work by testing whether fimepinostat targets DNA replication and replication stress response proteins in Ewing sarcoma cells.

**Figure 1:**
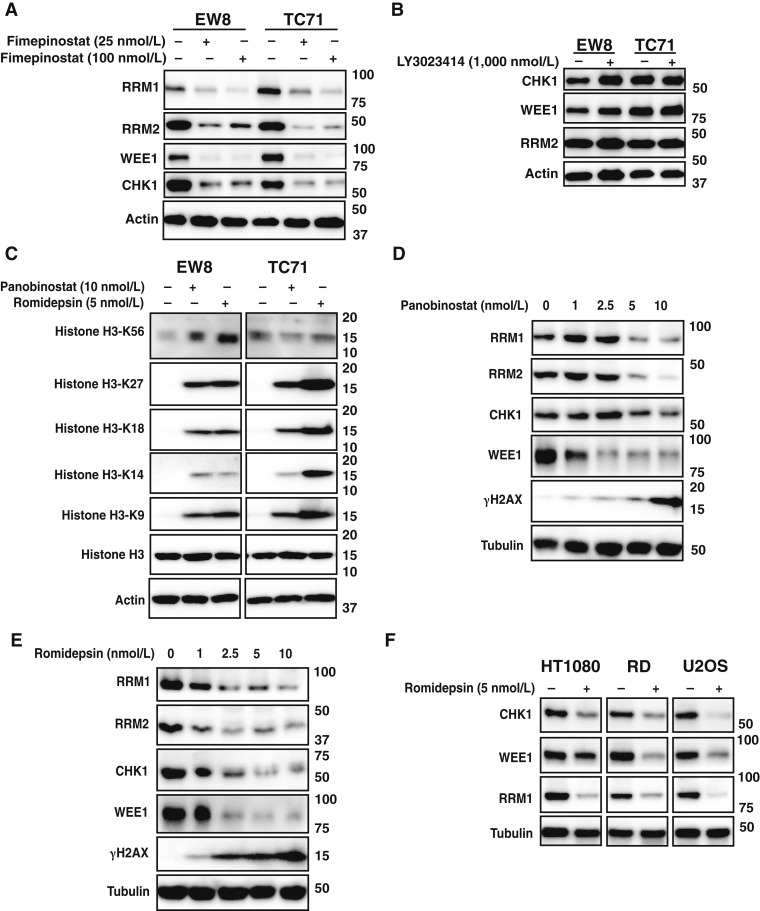
HDAC inhibitors decrease RRM1, RRM2, CHK1, and WEE1 protein levels. **A,** EW8 and TC71 cells were treated with fimepinostat (25 or 100 nmol/L) for 24 hours, and then cellular lysates were collected for immunoblotting. **B,** EW8 and TC71 cells were treated with LY3023414 (1,000 nmol/L) for 24 hours, and then cellular lysates were collected for immunoblotting. **C,** EW8 and TC71 cells were treated with panobinostat (10 nmol/L) or romidepsin (5 nmol/L) for 24 hours, and then cellular lysates were collected for immunoblotting. **D** and **E,** EW8 cells were treated with different doses of panobinostat (**D**) or romidepsin (**E**) for 24 hours, and then cellular lysates were collected for immunoblotting. **F,** Cell lines representing additional sarcoma subtypes were treated with romidepsin (5 nmol/L) for 24 hours, and then cellular lysates were collected for immunoblotting.

Figure 1A shows that the treatment of Ewing sarcoma cell lines, EW8 and TC71, with fimepinostat for 24 hours reduces the levels of RRM1, RRM2, CHK1, and WEE1. To determine whether this effect of fimepinostat was due to inhibition of HDAC, PI3K, or both targets, we treated the cell lines with the PI3K inhibitor LY3023414. Figure 1B demonstrates that LY3023414 did not alter the levels of the replication proteins, suggesting that HDAC inhibition is the more relevant target for regulation of the DNA replication and replication stress proteins. Next, we treated the Ewing sarcoma cell lines with low doses of panobinostat (pan-HDAC inhibitor) or romidepsin (an inhibitor of class 1 HDACs: HDAC1, HDAC2, HDAC3, and HDAC8) and assessed acetylation at multiple sites in the histone H3 protein. Figure 1C shows that both drugs, at doses of 5 to 10 nmol/L, increase histone acetylation. Dose–response experiments were then performed with both drugs to evaluate the effect of HDAC inhibition on RRM1, RRM2, CHK1, and WEE1 protein levels. Figure 1D and E demonstrate that panobinostat and romidepsin decrease the levels of RRM1, RRM2, CHK1, and WEE1 proteins while increasing the phosphorylation of γgH2AX, a marker of DNA damage.

Dose–response experiments demonstrated that HDAC inhibitors decrease Ewing sarcoma cell proliferation in 72-hour cell growth assays, consistent with the established role of these genes in cell survival and published work on the sensitivity of Ewing sarcoma cell lines to HDAC inhibitors (Supplementary Fig. S1; refs. 59–64). Ewing sarcoma cell lines were also previously shown to be sensitive to the CRISPR/Cas9-mediated knockout of class 1 HDACs, including HDAC1, HDAC2, and HDAC3 (59). Analysis of the Dependency Map (Broad Institute) data identified that the mRNA expression levels of HDAC1, HDAC2, and HDAC3, compared with class 2 HDACs, strongly correlate with the expression levels of the DNA replication genes (Supplementary Fig. S2A–S2C). We used siRNA to knock down individual class 1 HDACs but found that downregulation of one HDAC resulted in upregulation of other HDACs (Supplementary Fig. S3A). This compensatory response of class 1 HDACs was also reported in CRISPR/Cas9-mediated knockout experiments (59). Consequently, to further evaluate the effect of inhibiting multiple class 1 HDACs, we tested a second class 1 HDAC inhibitor, entinostat, and observed similar effects to romidepsin (Supplementary Fig. S3B). Finally, we treated additional sarcoma cell lines, including HT1080 (fibrosarcoma), RD (rhabdomyosarcoma), and U2OS (osteosarcoma), with romidepsin and observed a similar decrease in the levels of multiple replication proteins, demonstrating that the effect of HDAC inhibitors on DNA replication proteins is not limited to Ewing sarcoma tumors (Fig. 1F).

### HDAC inhibitors downregulate the expression of genes related to DNA replication and the cellular response to DNA replication stress

**Figure 2:**
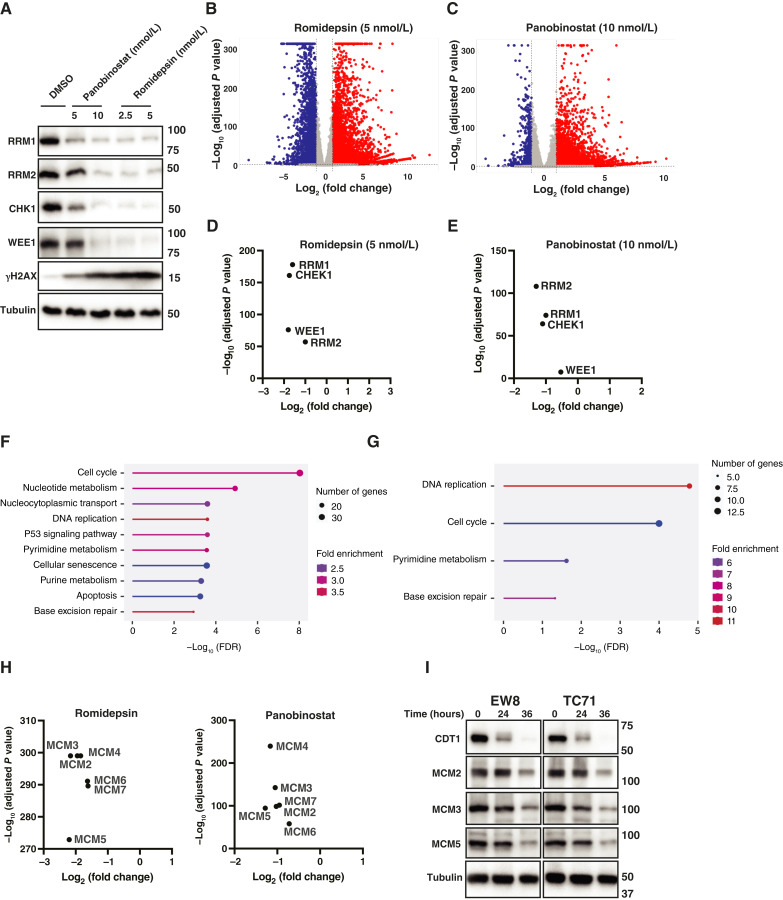
HDAC inhibitors downregulate the expression of genes related to DNA replication and the cellular response to DNA replication stress. **A,** EW8 cells were treated with panobinostat or romidepsin for 24 hours, and then cellular lysates and mRNA were collected for immunoblotting and RNA-seq analysis. **B** and **C,** Volcano plots of differentially expressed genes (fold change >2, adjusted *P* value < 0.05) in the EW8 cells treated with romidepsin (5 nmol/L; **B**) or panobinostat (10 nmol/L; **C**). **D–E,** RNA-seq data, fold change and adjusted *P* value, for the RRM1, RRM2, CHK1, and WEE1 genes in EW8 cells treated with romidepsin (5 nmol/L) and panobinostat (10 nmol/L). **F** and **G,** Gene sets (Kyoto Encyclopedia of Genes and Genomes pathway) enriched in the genes that are downregulated in EW8 cells treated with romidepsin (**F**) and panobinostat (**G**). **H,** RNA-seq data, fold change and adjusted *P* value, for the MCM2–7 genes in EW8 cells treated with romidepsin and panobinostat. **I,** EW8 and TC71 cells were treated with romidepsin (5 nmol/L) for 24 hours, and then cellular lysates were collected for immunoblotting.

Next, we investigated how HDAC proteins, which are epigenetic regulators of gene expression, regulate the levels of DNA replication proteins in Ewing sarcoma cells (65, 66). EW8 cells were treated with multiple doses of panobinostat (5 and 10 nmol/L) or romidepsin (2.5 and 5 nmol/L), which reduced levels of the DNA replication proteins, and then RNA-seq analysis was performed to assess the effect of HDAC inhibition on the transcriptome (Fig. 2A). Volcano plots for the two drugs show that the treatment of Ewing sarcoma cells with HDAC inhibitors results in more upregulated genes than downregulated genes, consistent with the well-established effect of histone acetylation on chromatin structure and accessibility (Fig. 2B and C; Supplementary Fig. S4; ref. 67). However, as shown in Fig. 2D and E, both HDAC inhibitors significantly downregulated the expression of the DNA replication–related genes RRM1, RRM2, CHK1, and WEE1. Moreover, gene set enrichment analysis, using the Kyoto Encyclopedia of Genes and Genomes pathway dataset, identified DNA replication as a top gene set downregulated by both drugs (Fig. 2F and G). Further analysis of the genes comprising these enriched gene sets unexpectedly identified that romidepsin and panobinostat also downregulate multiple components of the MCM2–7, which is the ATPase core of the pre-RC that licenses DNA replication origins (Fig. 2H; Supplementary Fig. S5; ref. 34). HDAC inhibitors decreased MCM2–7 expression by ∼2- to 4-fold (Fig. 2H), and Fig. 2I demonstrates that the levels of the MCM2–7 proteins are also decreased by treatment with romidepsin. Additionally, the level of CDT1, which loads the MCM2–7 complex onto DNA, was also decreased by treatment with an HDAC inhibitor. This effect on MCM2–7 and CDT1 expression is not a general response to DNA replication stress as previous RNA-seq analysis of EW8 and TC71 cell lines with conditional knockout and rescue of RRM1, which arrests DNA replication, did not show a similar effect on MCM2–7 or CDT1 mRNA levels (Supplementary Fig. S6; ref. 40).

### HDAC inhibitors impair DNA replication in Ewing sarcoma cells

**Figure 3:**
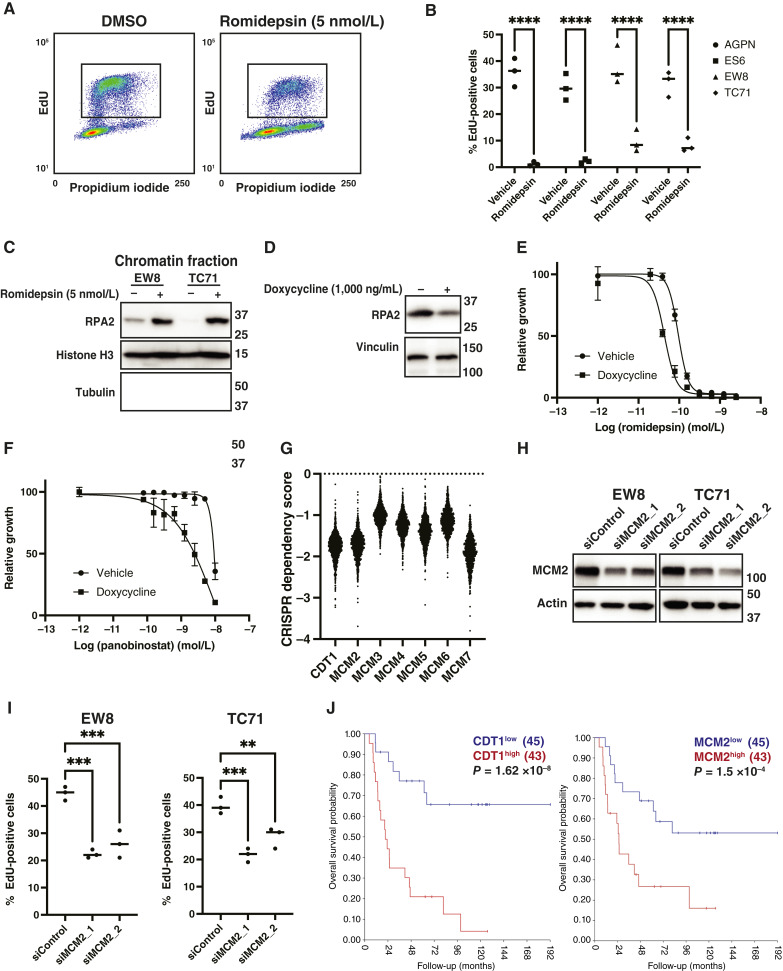
HDAC inhibitors impair DNA replication in Ewing sarcoma cells. **A,** Representative cell-cycle analysis (EdU and propidium iodide) plot for EW8 cells treated with romidepsin (5 nmol/L) for 24 hours. **B,** Summary of EdU incorporation efficiency for additional Ewing sarcoma cell lines treated with romidepsin (5 nmol/L) for 24 hours. **C,** Ewing sarcoma cell lines were treated with romidepsin (5 nmol/L) for 24 hours, and then chromatin-bound RPA2 was assessed using immunoblotting. **D,** EW8 cells expressing a doxycycline-inducible shRNA targeting RPA2 were treated with doxycycline for 24 hours, and then cellular lysates were collected for immunoblotting. **E** and **F,** EW8 cells expressing a doxycycline-inducible shRNA targeting RPA2 were treated with doxycycline (1,000 ng/mL) for 24 hours, and then dose-response experiments were performed with romidepsin (**E**) and panobinostat (**F**). Cell viability was measured 72 hours after drug addition using the alamarBlue assay. Error bars represent the mean ± SD of three technical replicates. The results are representative of two independent experiments. **G,** CRISPR dependency score for CDT1 and MCM2–7 (depmap.org). **H,** EW8 and TC71 cells were treated with two different siRNAs targeting MCM2, and then cellular lysates were collected for immunoblotting. **I,** EW8 and TC71 cells were treated with siRNAs targeting MCM2, and then EdU incorporation into DNA was measured using flow cytometry. **J,** Kaplan–Meier analysis shows overall survival of patients with Ewing sarcoma tumors according to the expression level of CDT1 and MCM2 mRNA. *P* values were calculated using a two-tailed Student *t* test or a one-way ANOVA followed by the Dunnett multiple comparisons test. **, *P* < 0.01; ***, *P* < 0.001; ****, *P* < 0.0001.

The downregulation of these pre-RC proteins is predicted to impair DNA replication and generate replication stress (33, 34). Figure 3A and B shows that romidepsin inhibits DNA replication, as assessed by measuring the incorporation efficiency of the thymidine analog EdU in multiple Ewing sarcoma cell lines. Moreover, DNA replication stress generates ssDNA, which is bound by RPA2, and Fig. 3C demonstrates that HDAC inhibitors increase chromatin-bound RPA2 (68). Reducing RPA2 levels using doxycycline-inducible shRNA also sensitizes Ewing sarcoma cells to romidepsin and panobinostat (Fig. 3D–F). Analysis of Dependency Map (Broad Institute) data identified that the loss of MCM2–7 proteins impairs proliferation in Ewing sarcoma and other cancer types (Fig. 3G; ref. 54). Similarly, knockdown of MCM2 using two different siRNAs inhibits DNA replication, as assessed using EdU labeling (Fig. 3H and I). Finally, high levels of CDT1 and the individual MCM2–7 proteins, which are associated with poor prognoses in multiple cancer types, are significantly associated with poor survival in patients with Ewing sarcoma tumors (Fig. 3J; Supplementary Fig. S7; refs. 69, 70).

### HDAC inhibitors upregulate and downregulate genes that are regulated by EWS::FLI1

**Figure 4:**
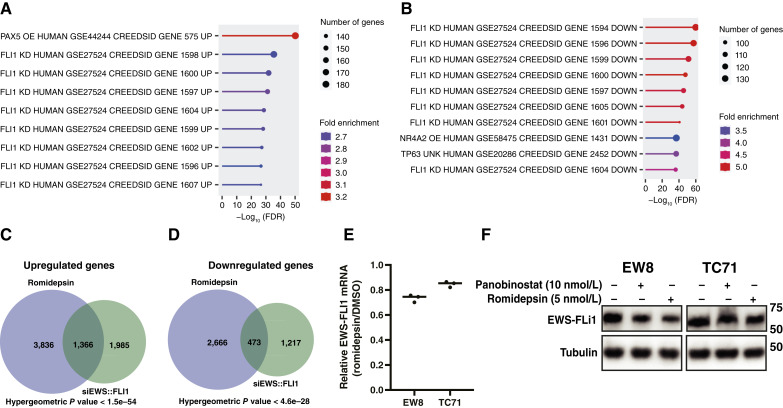
HDAC inhibitors upregulate and downregulate genes that are regulated by EWS::FLI1. **A** and **B,** Results of transcription factor enrichment analysis performed with the genes that are upregulated (**A**) and downregulated (**B**) in EW8 cells treated with romidepsin. **C** and **D,** Overlap between genes upregulated (**C**) and downregulated (**D**) in EW8 cells treated with romidepsin or siEWS::FLI1. **E,** Relative EWS::FLI1 mRNA in EW8 and TC71 cells treated with romidepsin for 24 hours. **F,** EW8 and TC71 cells were treated with romidepsin or panobinostat for 24 hours, and then cellular lysates were collected for immunoblotting for the EWS::FLI1 oncoprotein.

Next, we analyzed the gene expression data using transcription factor enrichment analysis and identified that HDAC inhibitors upregulate and downregulate genes that are also regulated by EWS::FLI1 (Fig. 4A and B). Previously, in published work, we used siRNA to knock down EWS::FLI1 and then performed RNA-seq analysis of the transcriptome (53). Supporting the transcription factor enrichment analysis, Fig. 4C and D demonstrates that HDAC inhibitors regulate a significant number of genes that are also regulated by EWS::FLI1 in the siRNA knockdown experiment. Although HDAC inhibitors have been reported to inhibit the expression of multiple oncogenes, including EWS::FLI1, we found that under the conditions tested, using low doses of HDAC inhibitors, EWS::FLI1 mRNA was not significantly altered in either cell line, and the EWS-FLI1 protein was only modestly decreased in one cell line (Fig. 4E and F; refs. 71, 72).

### HDAC inhibitors decrease BRD4 protein levels in Ewing sarcoma cells

**Figure 5:**
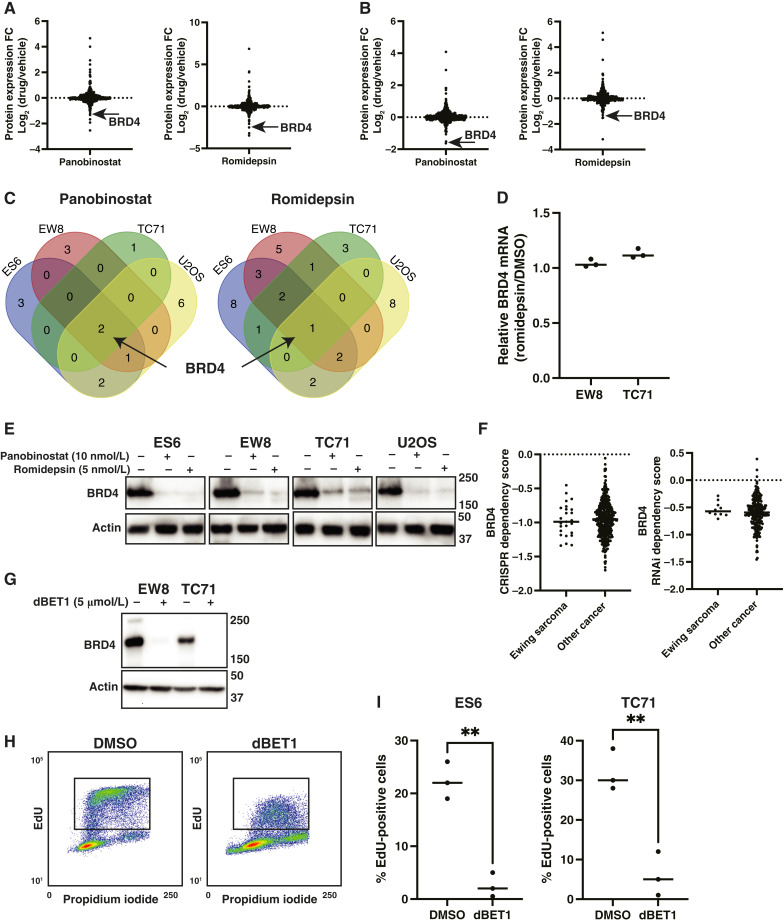
HDAC inhibitors decrease BRD4 protein levels in Ewing sarcoma cells. **A** and **B,** Comparison of protein expression, based on RPPAs, in EW8 (**A**) or TC71 (**B**) cells treated with romidepsin (5 nmol/L) or panobinostat (10 nmol/L) for 24 hours. **C,** Overlap of proteins downregulated >1.5-fold in the RPPA experiment by romidepsin and panobinostat in Ewing sarcoma (ES6, EW8, and TC71) and osteosarcoma (U2OS) cell lines. **D,** Relative BRD4 mRNA in EW8 and TC71 cells treated with romidepsin (5 nmol/L). **E,** EW8 and TC71 cells were treated with romidepsin or panobinostat for 24 hours, and then cellular lysates were collected for immunoblotting for BRD4. **F,** CRISPR and shRNA dependency scores for BRD4 (Dependency Map, Broad Institute) in Ewing sarcoma and other cancer cell lines. **G,** EW8 and TC71 cells were treated with dBET1 (5 μmol/L) for 18 hours, and then cellular lysates were collected for immunoblotting for BRD4. **H,** Representative cell-cycle analysis (EdU and propidium iodide) plot for EW8 cells treated with dBET1 (5 µmol/L) for 18 hours. **I,** Summary of EdU incorporation into DNA for additional Ewing sarcoma cell lines treated with dBET1 (5 µmol/L) for 18 hours. *P* values were calculated using a two-tailed Student *t* test. **, *P* < 0.01. FC, fold change.

Next, we performed RPPA analysis of Ewing sarcoma (EW8, TC71, and ES6) and U2OS (osteosarcoma) cell lines treated with panobinostat and romidepsin (Fig. 5A and B; Supplementary Fig. S8). Notably, a single protein, BRD4, was decreased >2-fold in all datasets (Fig. 5C; Supplementary Table S1). BRD4 is a transcriptional and epigenetic regulator that recognizes acetylated lysine residues (73). In addition, BRD4 is also a well-described coregulator of the EWS::FLI1 transcriptional program (43–45, 74). HDAC inhibitors did not decrease BRD4 mRNA, but immunoblotting confirmed the RPPA results, showing downregulation of BRD4 protein levels (Fig. 5D and E). BRD4 is reported to support cell proliferation in multiple cancer types, and analysis of Dependency Map (Broad Institute) data identified that reducing BRD4 levels, by shRNA knockdown or CRISPR knockout, inhibits the proliferation of Ewing sarcoma and other cancer cell lines (Fig. 5F). BRD4 is also reported to regulate DNA replication and the cellular response to DNA replication stress via multiple targets and mechanisms (75–77). Figure 5G–I demonstrate that decreasing BRD4 protein levels, using dBET1 (a small-molecule BRD4 degrader), inhibits DNA replication as assessed by EdU labeling of DNA.

### HDAC inhibitors downregulate survivin (BIRC5) in Ewing sarcoma cells

**Figure 6:**
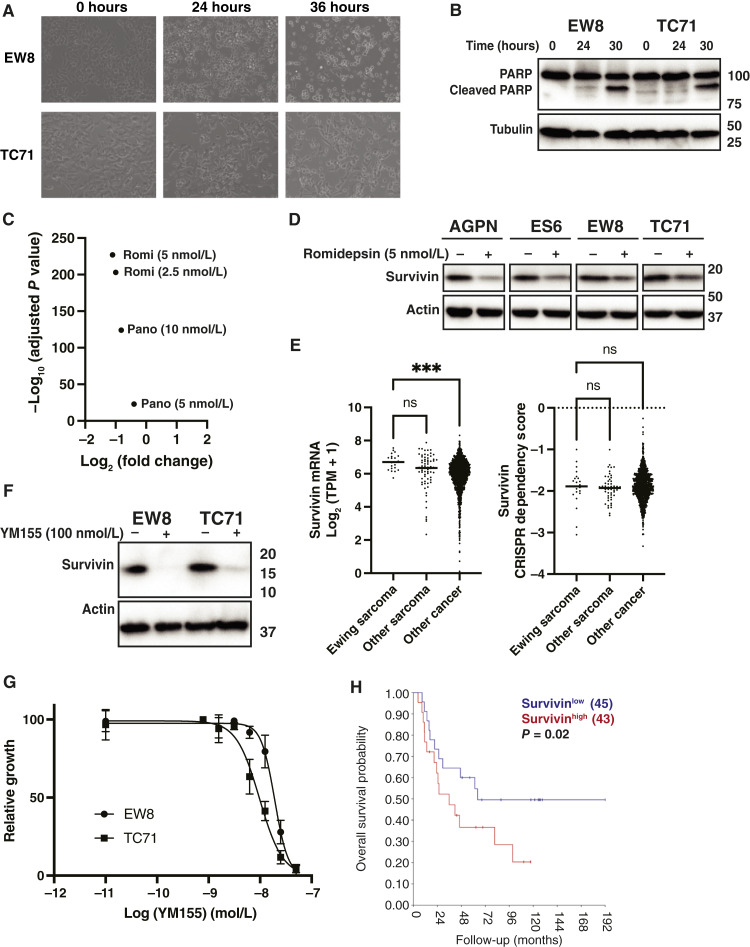
HDAC inhibitors downregulate survivin (BIRC5) in Ewing sarcoma cells. **A,** Treatment with romidepsin (5 nmol/L) causes morphologic changes in Ewing sarcoma cells. **B,** EW8 and TC71 cells were treated with romidepsin for 24 or 30 hours, and then cellular lysates were collected for immunoblotting. **C,** RNA-seq data, fold change and adjusted *P* value, for the survivin gene in EW8 cells treated with romidepsin and panobinostat. **D,** Ewing sarcoma cell lines were treated with romidepsin (5 nmol/L) for 24 hours, and then cellular lysates were collected for immunoblotting for survivin. **E,** Survivin mRNA expression level and CRISPR dependency score in Ewing sarcoma, other sarcoma, and other cancer cell lines. **F,** EW8 and TC71 cells were treated with YM155 for 24 hours, and then cellular lysates were collected for immunoblotting for survivin. **G,** EW8 cells were treated with different doses of YM115 for 72 hours, and then cell viability was measured using the alamarBlue assay. Error bars represent the mean ± SD of three technical replicates. The results are representative of two independent experiments. **H,** Kaplan–Meier analysis shows the overall survival of patients with Ewing sarcoma according to the expression level of survivin mRNA. *P* values were calculated using a one-way ANOVA followed by the Dunnett multiple comparisons test. TPM, transcripts per million. ***, *P* < 0.001.

Although our analysis focused on DNA replication, we expect that the effects of HDAC inhibitors affect additional pathways and targets beyond DNA replication. For example, gene set enrichment analysis (Hallmark dataset) of the genes downregulated by HDAC inhibitors identified enrichment for c-Myc targets. Immunoblotting validated that HDAC inhibitors downregulate the expression level of the c-Myc protein, which supports Ewing sarcoma tumorigenesis (Supplementary Fig. S9; refs. 78, 79). We also observed drug-induced changes in cell morphology suggestive of apoptosis (Fig. 6A), and Figure 6B shows that romidepsin treatment causes cleavage of PARP, a marker of apoptosis. Analysis of the RNA-seq data identified a ∼2-fold decrease in survivin (BIRC5), an antiapoptotic protein with a prosurvival function in Ewing sarcoma tumors (Fig. 6C; refs. 41, 42). Treatment of multiple Ewing sarcoma cell lines with romidepsin reduced the level of the survivin protein (Fig. 6D). Survivin is highly expressed in Ewing sarcoma cell lines, and the loss of survivin significantly decreases cell proliferation (Fig. 6E; refs. 41, 42, 80). Next, we treated EW8 cells with YM155, a drug that suppresses survivin expression (Fig. 6F), and identified a significant inhibition of cell growth (Fig. 6G). Finally, high levels of survivin are associated with poor survival in patients with Ewing sarcoma tumors (Fig. 6H; refs. 42, 80).

## Discussion

The EWS::FLI1 gene fusion is the driver mutation in most Ewing sarcoma tumors and functions, in part, as an aberrant transcription factor (81). EWS::FLI1 is an attractive therapeutic target in Ewing sarcoma tumors because it is required for tumorigenesis, but directly targeting this oncoprotein has been challenging despite significant efforts over the past three decades (82). A complementary therapeutic approach is to identify and target unique vulnerabilities incurred by the oncoprotein. Notably, our group and others have shown that Ewing sarcoma tumors are sensitive, *in vitro* and *in vivo* in xenograft tumors, to drugs targeting the DNA replication pathway, including drug combinations targeting RNR and the ATR–CHK1 signaling pathway (refs. 3, 17–29; bioRxiv 2024:2023.04.30.538578).

**Figure 7:**
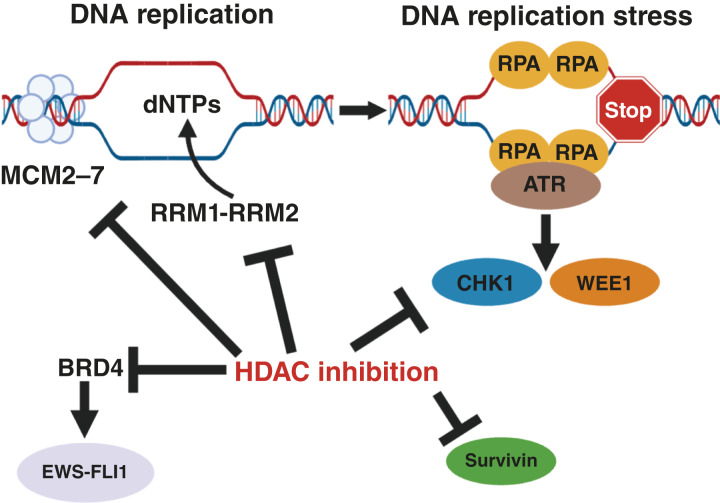
Integrated model for the regulation of DNA replication and replication stress response pathways by HDAC inhibitors. HDAC inhibitors downregulate the expression of multiple components of the pre-RC, including the MCM2–7 proteins and CDT1, as well as the RRM1 and RRM2 subunits of RNR, CHK1, and WEE1. HDAC inhibitors also downregulate the levels of the BRD4 and survivin proteins.

In this study, we identified that HDAC inhibitors decrease the mRNA and protein levels of multiple proteins, including RRM1, RRM2, CHK1, and WEE1, that have critical roles in DNA replication and the response to DNA replication stress (Fig. 7). Unexpectedly, our RNA-seq and proteomic analyses also revealed that HDAC inhibitors decrease the expression of the pre-RC components MCM2–7 and CDT1, as well as BRD4, a transcriptional cofactor for the *EWS::FLI1* oncoprotein (Fig. 7).

The strategy of targeting the pre-RC to enhance the efficacy of drugs targeting DNA replication is unexplored compared with efforts that have focused on understanding how the DNA damage response kinases, including ATR and CHK1, mediate resistance to drugs that cause or exacerbate DNA replication stress. Moreover, translational methods to target the MCM2–7 proteins in the clinic are lacking (30, 31, 70). However, in this work, we identified that HDAC inhibitors downregulate the expression of the MCM2–7 proteins, as well as the CDT1 protein that loads MCM2–7 onto the DNA. As MCM2–7 is loaded onto DNA in late mitosis and the G_1_ phase of the cell cycle, HDAC inhibitors may provide a novel approach to preemptively sensitize cells to DNA replication stress and, thereby, target DNA replication in cells before DNA replication begins (30, 35, 83).

Further supporting a critical role for the MCM2–7 and the pre-RC in Ewing sarcoma tumors, the Dbf4-dependent Cdc7 kinase (DDK), which initiates replisome assembly by phosphorylating the N-terminal tails of Mcm2, Mcm4, and Mcm6, was recently identified as a target in Ewing sarcoma tumors (3, 26, 27). Treatment of Ewing sarcoma cell lines with DDK inhibitors reduced the rate of DNA replication, prolonged the S-phase, induced aberrant mitotic progression, and demonstrated synergy with WEE1 inhibitors (3, 26, 27). In future work, we plan to test for synergy between HDAC and DDK inhibitors, as well as assess the effects of the drug combination on replisome function.

HDAC inhibitors are known to downregulate the expression of multiple oncogenes, including EWS::FLI1 (71, 72). The effects of HDAC inhibitors on the transcription of the EWS::FLI1 oncogene are dose-dependent with downregulation of EWS::FLI1 occurring at higher drug doses (71). In our experiments, using low doses of HDAC inhibitors (<10 nmol/L), we did not observe an effect of HDAC inhibitors on EWS::FLI1 levels. However, we expect that the modulation of EWS::FLI1 levels by HDAC inhibitors will be dependent on multiple factors, including the specific cell line, HDAC inhibitor, duration of treatment, and drug dose. Although low-dose HDAC inhibitors did not affect the level of EWS::FLI1 in our experiments, proteomic analyses identified that HDAC inhibitors do reduce the level of the BRD4 protein in Ewing sarcoma cell lines. BRD4 is a transcriptional regulator that recognizes acetylated lysine residues and functions as a coregulator for the transcriptional program of the EWS::FLI1 oncoprotein (43–45, 74). Thus, the loss of BRD4 is expected to contribute to the overlap in gene expression signatures that we identified between HDAC inhibitors and the shRNA-mediated downregulation of EWS::FLI1.

HDAC inhibition is also reported to increase histone 4 polyacetylation, which is a preferred binding substrate of BRD4, and retarget BRD4 to the bodies of actively transcribed genes (84). This redistribution of BRD4 could further disrupt the function of BRD4 in Ewing sarcoma cells and exacerbate the effects of HDAC inhibitors on the decrease of BRD4 protein levels. Moreover, BRD4 has also been reported to regulate multiple steps of DNA replication and the response to DNA replication stress, including activation of the ATR–CHK1 pathway and resolution of R-loops. R-loops, which are upregulated in Ewing sarcoma tumors, are DNA–RNA hybrids that impair DNA replication and cause DNA replication stress (75–77). We hypothesize that the loss of BRD4 mediated by HDAC inhibitors could further upregulate R-loops in Ewing sarcoma tumors, which will be tested in future work. In addition, recent work has also identified that inhibition of HDAC8 activity can upregulate R-loops in cancer cells (85).

HDAC inhibitors exhibit pleiotropic effects in cancer cells, targeting diverse pathways that are critical for tumorigenesis (59, 62–64). Although our study focused on the effects of HDAC inhibitors on DNA replication, we also identified additional targets and pathways, including survivin and c-Myc, that support tumorigenesis in Ewing sarcoma tumors and are regulated by HDAC inhibitors. Elucidating these additional pathways will be crucial for identifying drugs that synergize with HDAC inhibitors and advancing drug combinations to the clinic. For example, recent work identified that Ewing sarcoma tumors are highly sensitive to kt-3283, a bifunctional PARP-HDAC inhibitor (60). PARP proteins respond to DNA damage, including damage caused by DNA replication stress, and we hypothesize that the effects of HDAC inhibitors on DNA replication could contribute to the sensitivity of Ewing sarcoma tumors to dual inhibition of HDAC and PARP (86, 87). Similarly, panobinostat was recently shown to improve the efficacy of standard-of-care drugs, including doxorubicin and etoposide, in targeting Ewing sarcoma cells (61).

HDACs comprise a group of 18 enzymes that are divided into four classes. We found that panobinostat, a pan-HDAC inhibitor, and romidepsin, a class I inhibitor, are both effective in reducing the levels of DNA replication–related proteins (65, 66, 88, 89). Future work will focus on dissecting the contributions of specific HDACs, with an initial focus on the class 1 enzymes (HDAC1, HDAC2, HDAC3, and HDAC8) due to the sensitivity of Ewing sarcoma cell lines to the class 1 HDAC inhibitors romidepsin and entinostat, as well as a published CRISPR screen that identified the sensitivity of Ewing sarcoma cell lines to the loss of class 1 HDACs (59, 61). However, due to the compensatory response of class 1 HDACs to the knockdown of individual HDACs, as well as the correlation between the expression of HDAC1, HDAC2, and HDAC3 and replication genes, we expect that multiple HDACs may contribute to the regulation of the replication genes. In addition, our current work did not distinguish whether HDACs regulate the expression of DNA replication genes by a direct mechanism, via modification of histone acetylation at these specific genes, or an indirect mechanism, such as regulation of upstream transcription factor(s). This question will also be investigated in future work.

Our study focused on the effects of HDAC inhibitors on DNA replication but did not evaluate their impact on Ewing sarcoma tumor growth *in vivo* using xenograft models. However, previous studies have shown that HDAC inhibitors can reduce the growth of Ewing sarcoma xenografts in mice (59, 63, 90). Future work will assess whether the *in vitro* mechanisms related to DNA replication observed in this study also apply *in vivo*. Several HDAC inhibitors—romidepsin, vorinostat, and belinostat—are currently FDA-approved for treating hematologic malignancies such as multiple myeloma and peripheral T-cell lymphoma (91). Panobinostat was previously approved by the FDA for the treatment of multiple myeloma, but this approval was withdrawn due to a lack of completion of postapproval clinical studies. A case report described an adult with progressive, metastatic Ewing sarcoma who was treated with panobinostat and experienced prolonged disease stabilization for 18 months (92). Despite these findings, HDAC inhibitors have shown limited efficacy in solid tumors when used as single agents, prompting the investigation of combination therapies in clinical trials (91). Several early-phase clinical trials have tested HDAC inhibitors in children, mostly as single agents (93–99). Notably, vorinostat has been evaluated in combination with temozolomide in children (93, 94). Additionally, the Children’s Oncology Group New Agents for Rhabdomyosarcoma Task Force identified HDAC inhibitors as a priority class of drugs for further investigation (100). For aggressive sarcomas, such as Ewing sarcoma, combination drug therapy will likely be necessary. Understanding how HDAC inhibitors target Ewing sarcoma cells will aid in identifying synergistic drug partners.

In summary, our work demonstrates that HDACs regulate DNA replication and the response to DNA replication stress through multiple mechanisms. We provide evidence that HDAC inhibitors downregulate the expression of multiple components of the pre-RC, including the MCM2–7 proteins and CDT1, as well as the RRM1 and RRM2 subunits of RNR, CHK1, and WEE1. Additionally, we identified that HDAC inhibitors decrease the expression of the BRD4 protein, a bromodomain and extraterminal domain (BET) protein that regulates both the transcriptional program of the EWS::FLI1 oncoprotein and DNA replication. Overall, these results offer novel insights into the mechanism by which HDAC inhibitors target cancer cells and DNA replication.

## Supplementary Material

Figure S1 Romidepsin and panobinostat inhibit the growth of Ewing sarcoma cells.

Figure S2 Expression of HDAC1, HDAC2, and HDAC3 correlate with expression of RRM1, RRM2, CHEK1, and WEE1.

Figure S3 siRNA-mediated knockdown of HDAC1, 2, 3, and 8.

Figure S4 HDAC inhibitors down-regulate the levels of the RRM1, RRM2, CHK1, and WEE1 proteins.

Figure S5 Genes, which are downregulated in EW8 cells treated with romidepsin, that are enriched in the Kegg Pathway DNA replication gene set.

Figure S6 Effect of DNA replication arrest on MCM2-7 gene expression.

Figure S7 Kaplan-Meier analysis shows overall survival of patients with Ewing sarcoma tumors according to expression level of MCM3-7 mRNA.

Figure S8 Effects of romidepsin and panobinostat on protein expression levels.

Figure S9 Romidepsin and panobinostat downregulate the level of the c-Myc protein.

Supplementary Table 1 Reverse phase protein array (RPPA) data for cell lines treated with romidepsin or panobinostat compared to vehicle treatment.

Supplementary Data Full and unedited blots
