# Supplementary material for: Histone Deacetylase Inhibitors Target DNA Replication Regulators and Replication Stress in Ewing Sarcoma Cells
Source: Cancer Res Commun. 2025 Jun 27;5(6):1034–48. doi: 10.1158/2767-9764.CRC-25-0058 (PMC12202856; doi:10.1158/2767-9764.CRC-25-0058)
Supplement: Figure S1 — Romidepsin and panobinostat inhibit the growth of Ewing sarcoma cells. [file crc-25-0058_figure_s1_suppsf1.pdf]

## Supplemental Figure 1

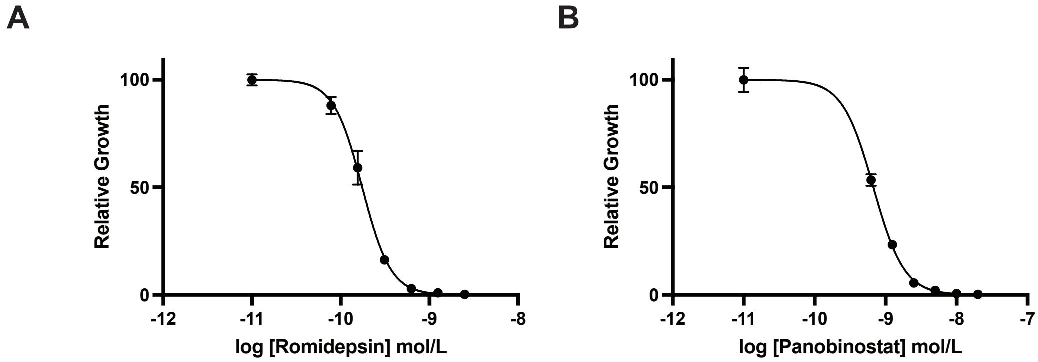


**Supplemental Figure 1.** Romidepsin and panobinostat inhibit the growth of Ewing sarcoma cells. (A) EW8 cells were treated with different doses of romidepsin for 72 hours and then cell viability was measured using the AlamarBlue assay. Error bars represent the mean ± SD of three technical replicates. (B) EW8 were treated with different doses of panobinostat for 72 hours and then cell viability was measured using the AlamarBlue assay. Error bars represent the mean ± SD of three technical replicates.
