# Supplementary material for: Histone Deacetylase Inhibitors Target DNA Replication Regulators and Replication Stress in Ewing Sarcoma Cells
Source: Cancer Res Commun. 2025 Jun 27;5(6):1034–48. doi: 10.1158/2767-9764.CRC-25-0058 (PMC12202856; doi:10.1158/2767-9764.CRC-25-0058)
Supplement: Figure S2 — Expression of HDAC1, HDAC2, and HDAC3 correlate with expression of RRM1, RRM2, CHEK1, and WEE1. [file crc-25-0058_figure_s2_suppsf2.pdf]

Supplemental Figure 2

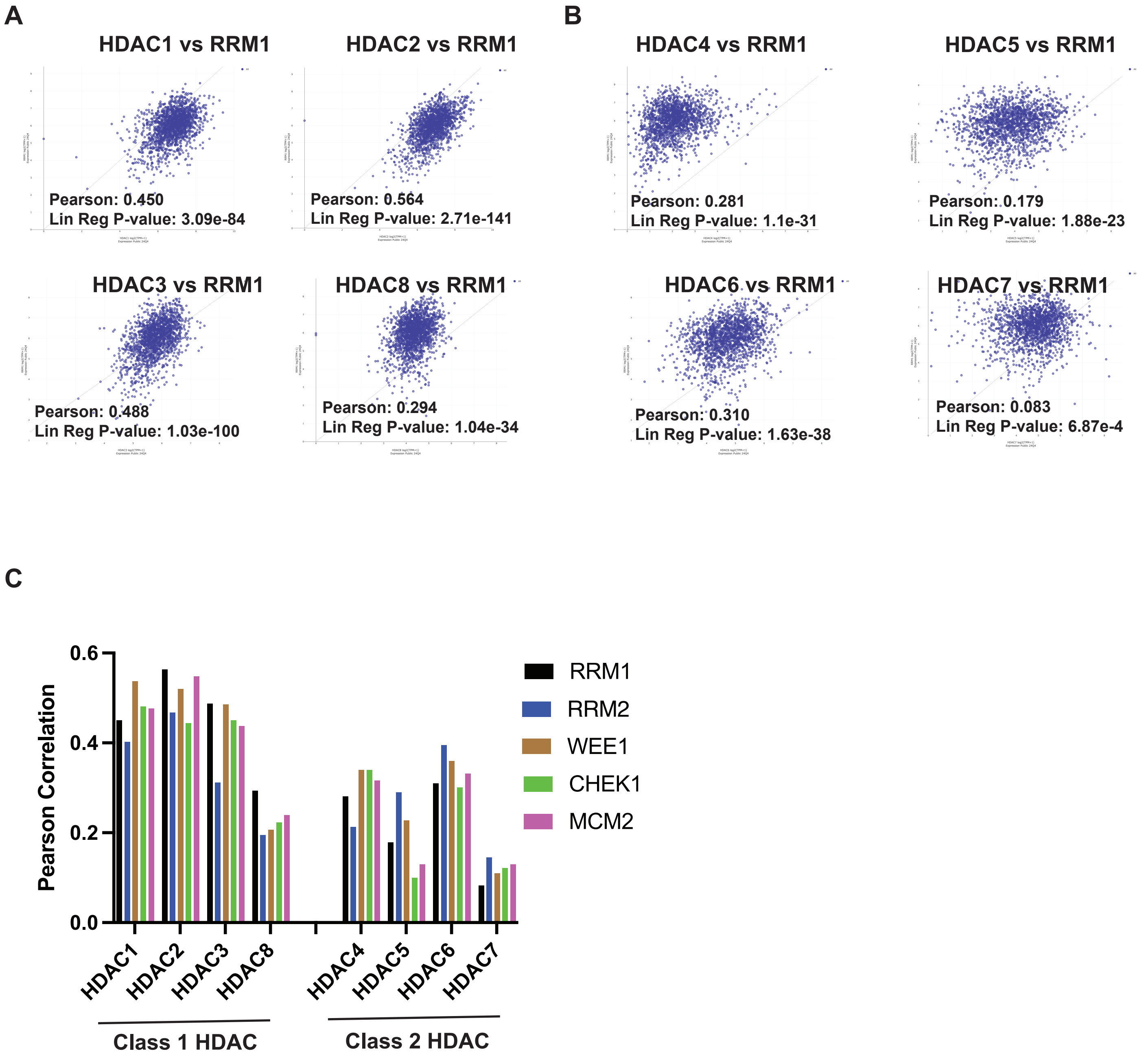

Supplemental Figure 2. Expression of HDAC1, HDAC2, and HDAC3 correlate with expression of RRM1, RRM2, CHEK1, and WEE1. (A) Correlation between mRNA expression for class 1 HDACs (HDAC1, 2, 3, and 8) and mRNA expression for RRM1 in cancer cell lines (Dependency Map; Broad Institute). (B) Correlation between mRNA expression for class 2 HDACs (HDAC4, 5, 6, and 7) and mRNA expression for RRM1 in cancer cell lines (Dependency Map; Broad Institute). (C) Summary data of Pearson correlations for class 1 and 2 HDACs and RRM1, RRM2, CHEK1, and WEE1 for cancer cell lines (Dependency Map; Broad Institute).
