# Supplementary material for: Histone Deacetylase Inhibitors Target DNA Replication Regulators and Replication Stress in Ewing Sarcoma Cells
Source: Cancer Res Commun. 2025 Jun 27;5(6):1034–48. doi: 10.1158/2767-9764.CRC-25-0058 (PMC12202856; doi:10.1158/2767-9764.CRC-25-0058)
Supplement: Figure S3 — siRNA-mediated knockdown of HDAC1, 2, 3, and 8. [file crc-25-0058_figure_s3_suppsf3.pdf]

Supplemental Figure 3

A

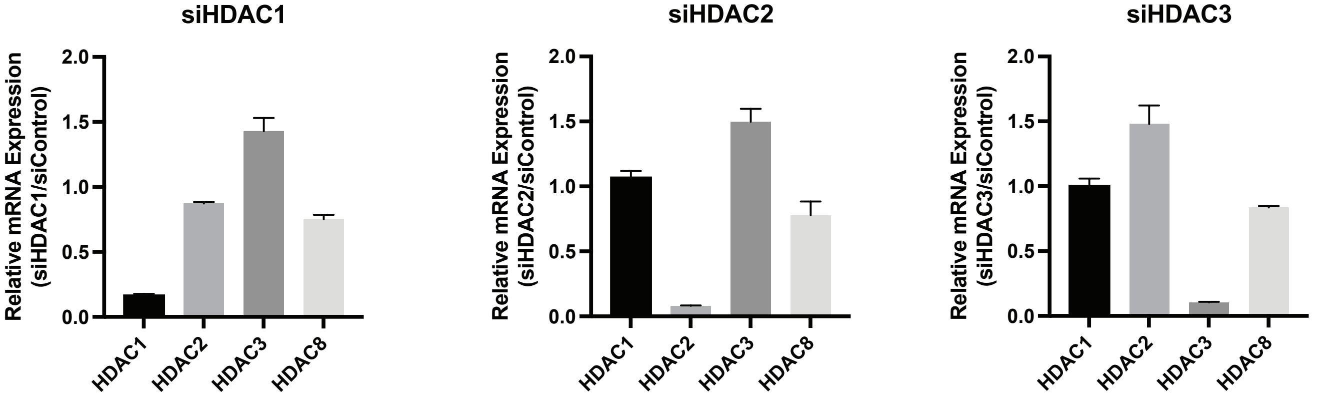

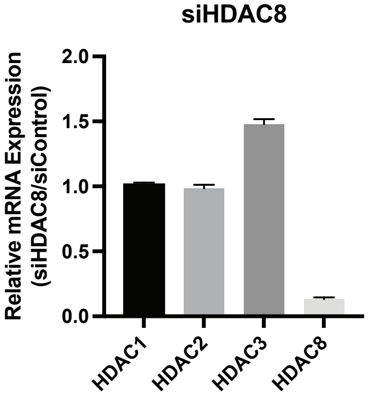

B

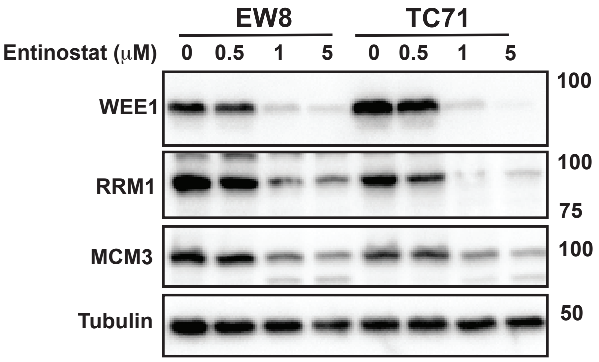

Supplemental Figure 3. siRNA-mediated knockdown of HDAC1, 2, 3, and 8. (A) EW8 cells were treated with siRNA targeting HDAC1, 2, 3, and 8 or a non-targeting siRNA for 48 hours. mRNA was then collected for RT-qPCR. (B) EW8 and TC71 cells were treated with entinostat for 24 hours and then cellular lysates were collected for immunoblotting.
