# Supplementary material for: Histone Deacetylase Inhibitors Target DNA Replication Regulators and Replication Stress in Ewing Sarcoma Cells
Source: Cancer Res Commun. 2025 Jun 27;5(6):1034–48. doi: 10.1158/2767-9764.CRC-25-0058 (PMC12202856; doi:10.1158/2767-9764.CRC-25-0058)
Supplement: Figure S4 — HDAC inhibitors down-regulate the levels of the RRM1, RRM2, CHK1, and WEE1 proteins. [file crc-25-0058_figure_s4_suppsf4.pdf]

Supplemental Figure 4

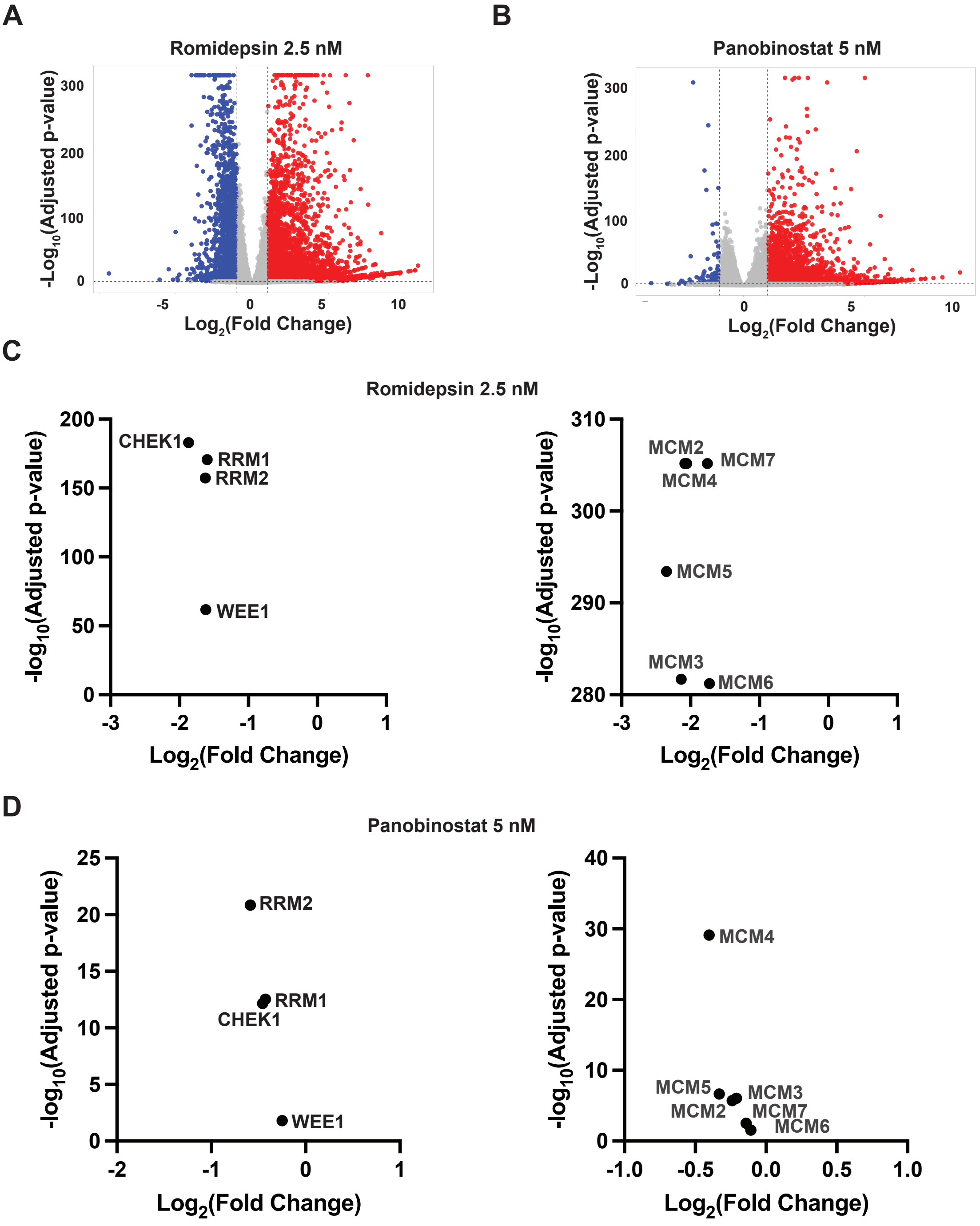

Supplemental Figure 4. HDAC inhibitors down-regulate the levels of the RRM1, RRM2, CHK1, and WEE1 proteins. (A-B) EW8 cells were treated with panobinostat (5 nM) or romidepsin (2.5 nM) for 24 hours and then cellular lysates and mRNA were collected RNA-seq analysis. Volcano plots of differentially expressed genes (Fold >2, adjusted P-value <0.05) in the EW8 cells treated with romidepsin (A) or panobinostat (B). (C-D) RNA-seq data, fold change and adjusted P-value, for the RRM1, RRM2, CHK1, WEE1, and MCM2-7 genes in EW8 cells treated with romidepsin (C) and panobinostat (D).
