# Supplementary material for: Histone Deacetylase Inhibitors Target DNA Replication Regulators and Replication Stress in Ewing Sarcoma Cells
Source: Cancer Res Commun. 2025 Jun 27;5(6):1034–48. doi: 10.1158/2767-9764.CRC-25-0058 (PMC12202856; doi:10.1158/2767-9764.CRC-25-0058)
Supplement: Figure S5 — Genes, which are downregulated in EW8 cells treated with romidepsin, that are enriched in the Kegg Pathway DNA replication gene set. [file crc-25-0058_figure_s5_suppsf5.pdf]

Supplemental Figure 5

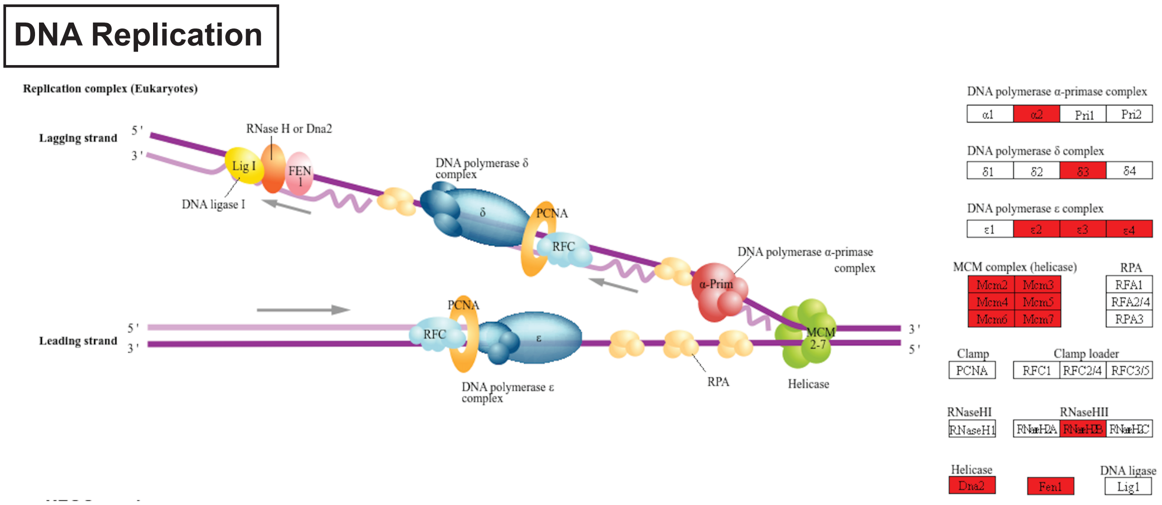

Supplemental Figure 5. Genes, which are downregulated in EW8 cells treated with romidepsin, that are enriched in the Kegg Pathway DNA replication gene set.
