# Supplementary material for: Histone Deacetylase Inhibitors Target DNA Replication Regulators and Replication Stress in Ewing Sarcoma Cells
Source: Cancer Res Commun. 2025 Jun 27;5(6):1034–48. doi: 10.1158/2767-9764.CRC-25-0058 (PMC12202856; doi:10.1158/2767-9764.CRC-25-0058)
Supplement: Figure S6 — Effect of DNA replication arrest on MCM2-7 gene expression. [file crc-25-0058_figure_s6_suppsf6.pdf]

Supplemental Figure 6

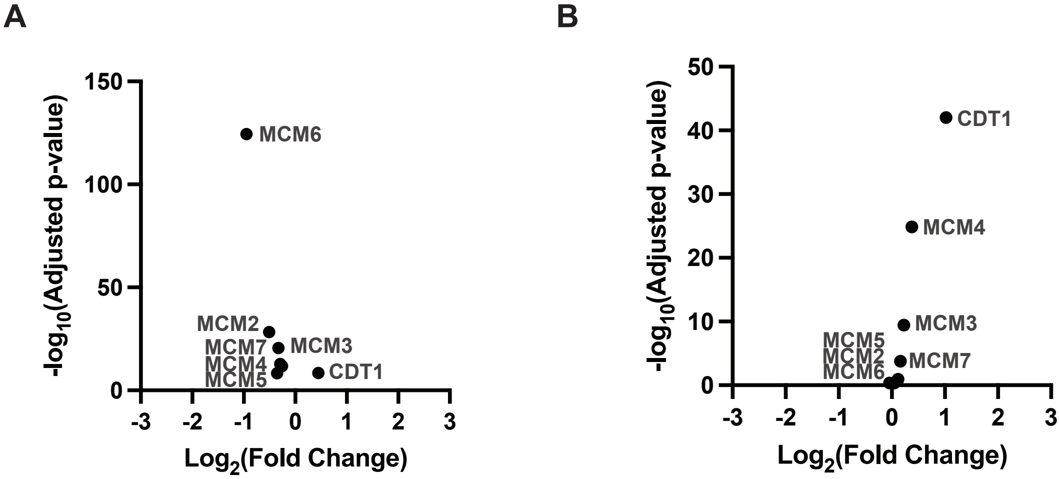

Supplemental Figure 6. Effect of DNA replication arrest on MCM2-7 gene expression. (A-B) RNA-seq data, fold change and adjusted P-value, for the RRM1, RRM2, CHK1, WEE1, and MCM2-7 genes in EW8 (A) and TC71 (B) cells with CRISPR-mediated, conditional knockout and rescue of RRM1, which arrests DNA replication.
