# Supplementary material for: Histone Deacetylase Inhibitors Target DNA Replication Regulators and Replication Stress in Ewing Sarcoma Cells
Source: Cancer Res Commun. 2025 Jun 27;5(6):1034–48. doi: 10.1158/2767-9764.CRC-25-0058 (PMC12202856; doi:10.1158/2767-9764.CRC-25-0058)
Supplement: Figure S7 — Kaplan-Meier analysis shows overall survival of patients with Ewing sarcoma tumors according to expression level of MCM3-7 mRNA. [file crc-25-0058_figure_s7_suppsf7.pdf]

Supplemental Figure 7

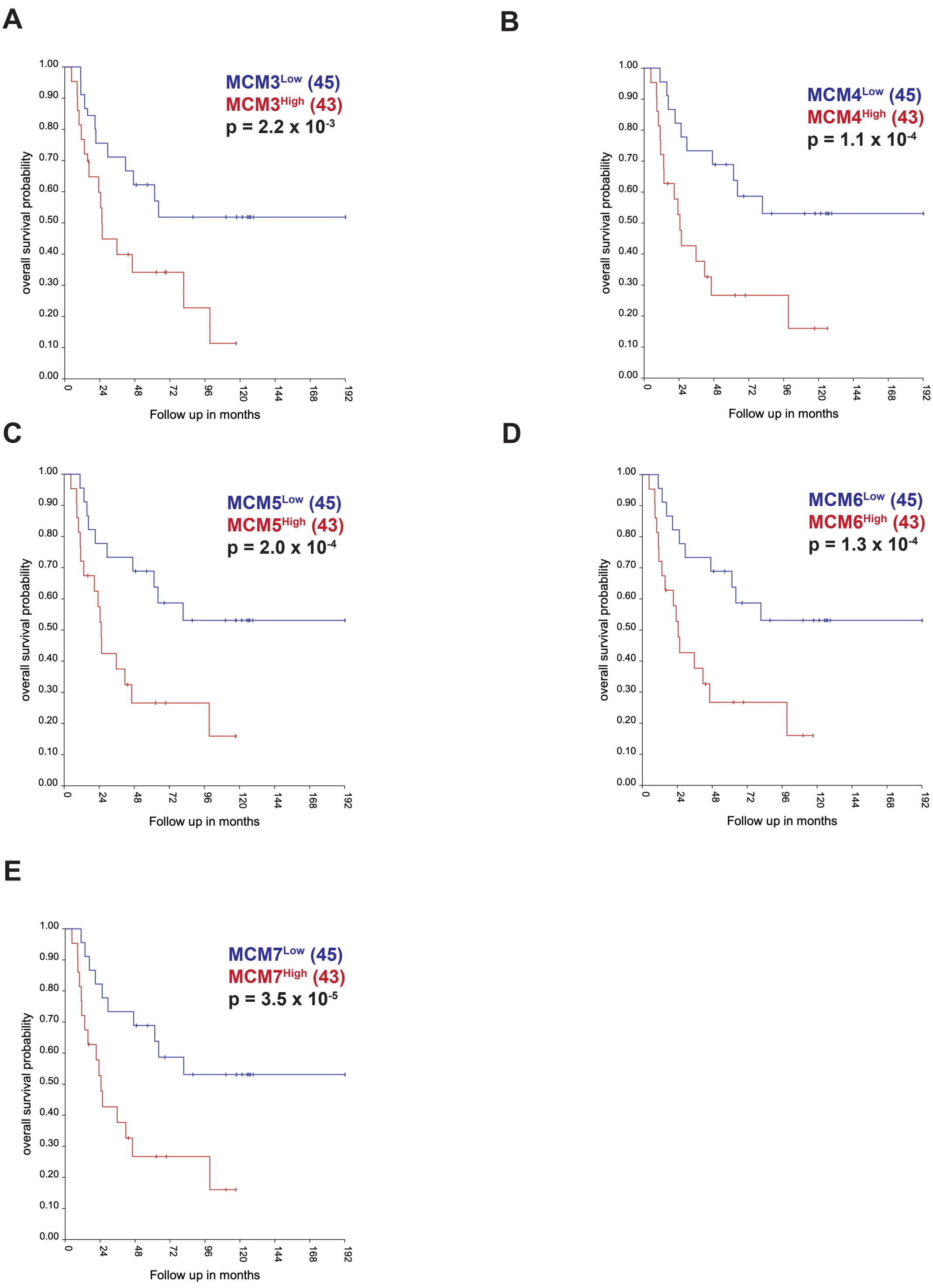

Supplemental Figure 7. Kaplan-Meier analysis shows overall survival of patients with Ewing sarcoma tumors according to expression level of MCM3-7 mRNA.
