# Supplementary material for: Histone Deacetylase Inhibitors Target DNA Replication Regulators and Replication Stress in Ewing Sarcoma Cells
Source: Cancer Res Commun. 2025 Jun 27;5(6):1034–48. doi: 10.1158/2767-9764.CRC-25-0058 (PMC12202856; doi:10.1158/2767-9764.CRC-25-0058)
Supplement: Figure S8 — Effects of romidepsin and panobinostat on protein expression levels. [file crc-25-0058_figure_s8_suppsf8.pdf]

## Supplemental Figure 8

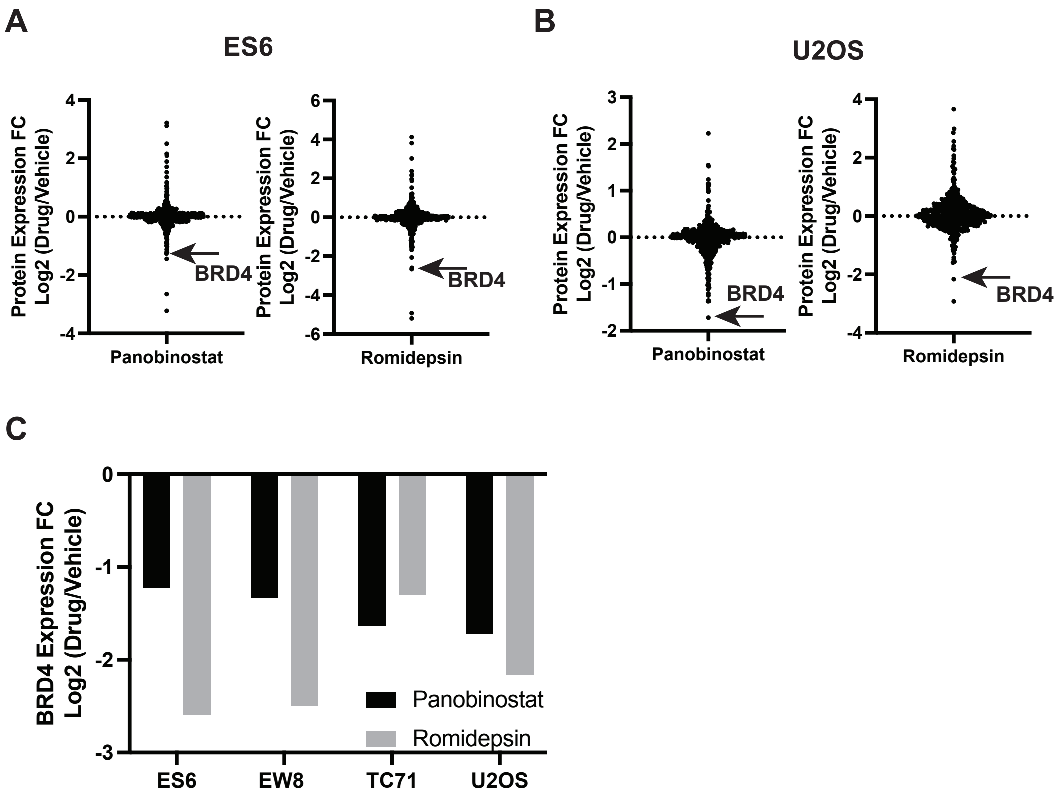


**Supplemental Figure 8.** Effects of romidepsin and panobinostat on protein expression levels. (A-B) Comparison of protein expression, based on reverse phase protein arrays (RPPA), in ES6 (A) or U2OS (B) cells treated with romidepsin (5 nM) or panobinostat (10 nM) for 24 hours. (C) Log2Fold change in BRD4 protein levels in cell lines treated with romidepsin or panobinostat.
