# Supplementary material for: Histone Deacetylase Inhibitors Target DNA Replication Regulators and Replication Stress in Ewing Sarcoma Cells
Source: Cancer Res Commun. 2025 Jun 27;5(6):1034–48. doi: 10.1158/2767-9764.CRC-25-0058 (PMC12202856; doi:10.1158/2767-9764.CRC-25-0058)
Supplement: Figure S9 — Romidepsin and panobinostat downregulate the level of the c-Myc protein. [file crc-25-0058_figure_s9_suppsf9.pdf]

Supplemental Figure 9

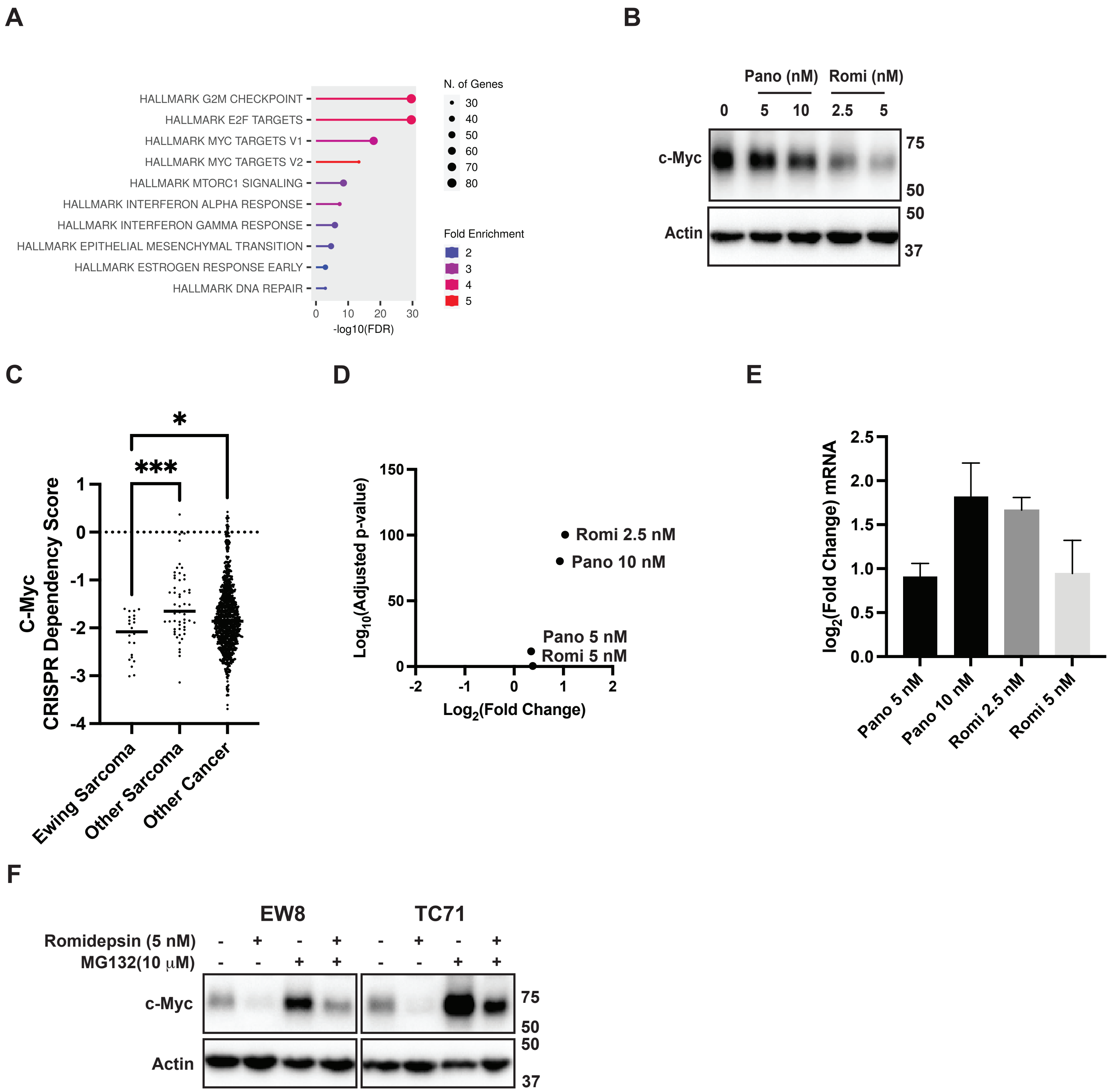

Supplemental Figure 9. Romidepsin and panobinostat downregulate the level of the c-Myc protein. (A) Gene set enrichment analysis (Hallmark set) for genes downregulated by romidepsin. (B) EW8 cells were treated with panobinostat or romidepsin for 24 hours and then cellular lysates were collected for immunoblotting for c-Myc. (C) CRISPR dependency scores for BRD4 (Dependency Map, Broad Institute) in Ewing sarcoma and other cancer cell lines. (D) RNA-seq data, fold change and adjusted P-value, for the c-Myc gene in EW8 cells treated with romidepsin or panobinostat. (E) RT-qPCR for c-MYC mRNA in EW8 cells treated with romidepsin or panobinostat for 24 hours. (F) EW8 and TC71 cells were treated with romidepsin for 18 hours and then MG132 (proteasome inhibitor) was added for an additional 6 hours. Cellular lysates were then collected for immunoblotting for c-Myc.
