# Supplementary material for: Histone Deacetylase Inhibitors Target DNA Replication Regulators and Replication Stress in Ewing Sarcoma Cells
Source: Cancer Res Commun. 2025 Jun 27;5(6):1034–48. doi: 10.1158/2767-9764.CRC-25-0058 (PMC12202856; doi:10.1158/2767-9764.CRC-25-0058)

Full unedited blots for Figure 1A-B

A

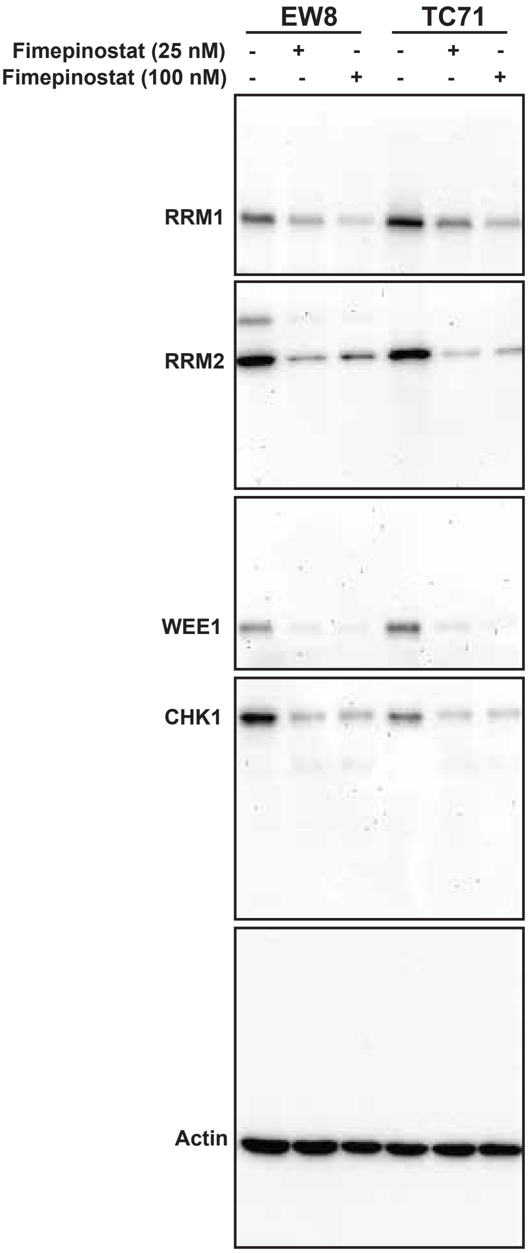

B

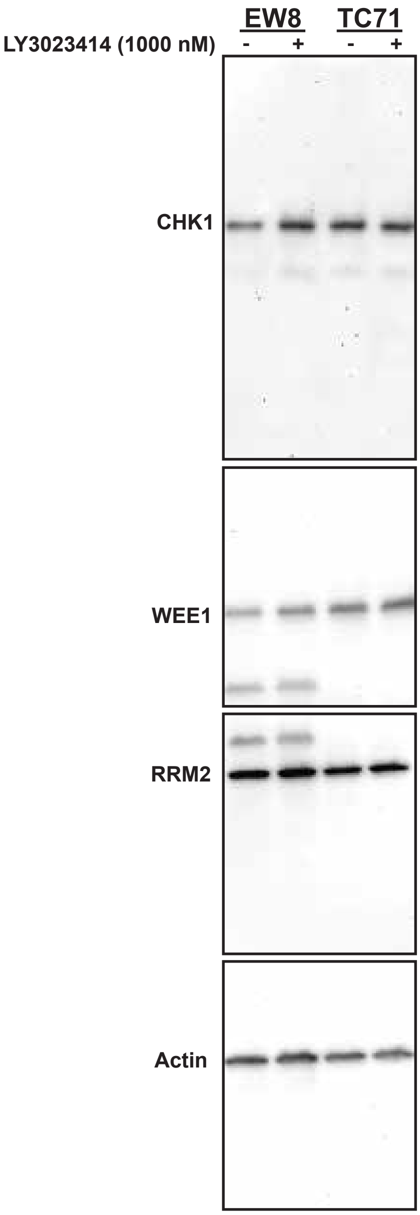

Full unedited blots for Figure 1C

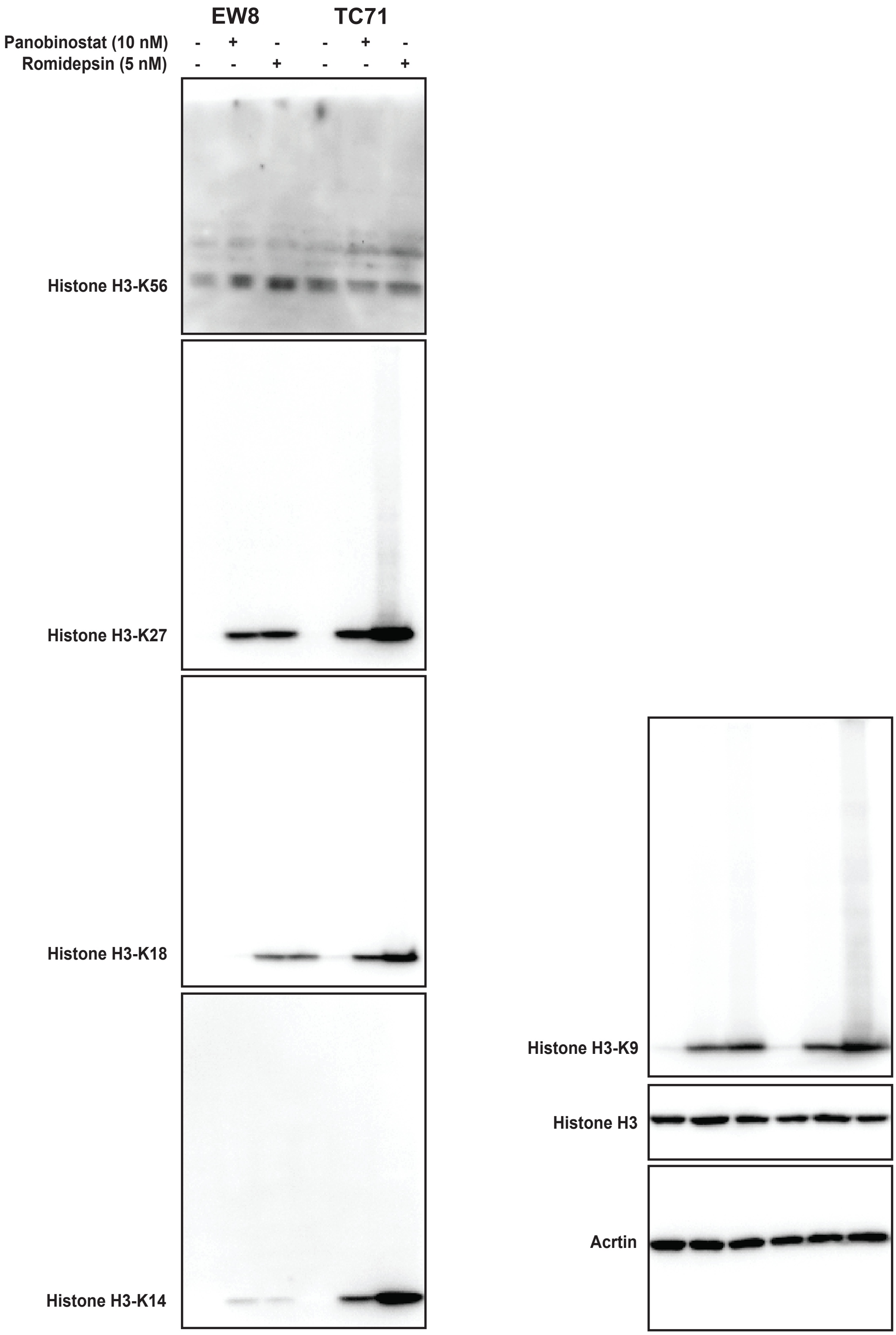

Full unedited blots for Figure 1D-F

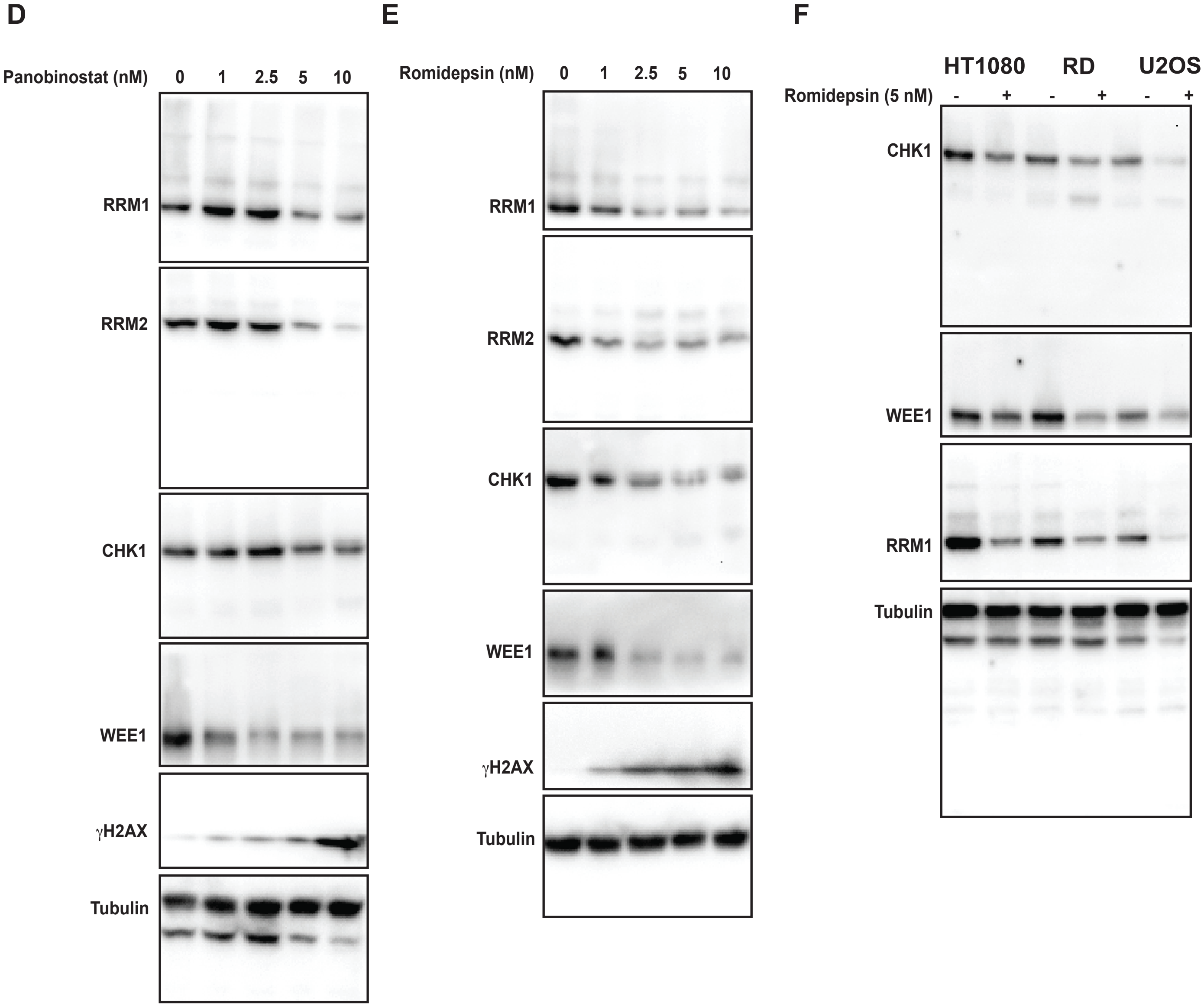

Full and unedited blot for Figure 2

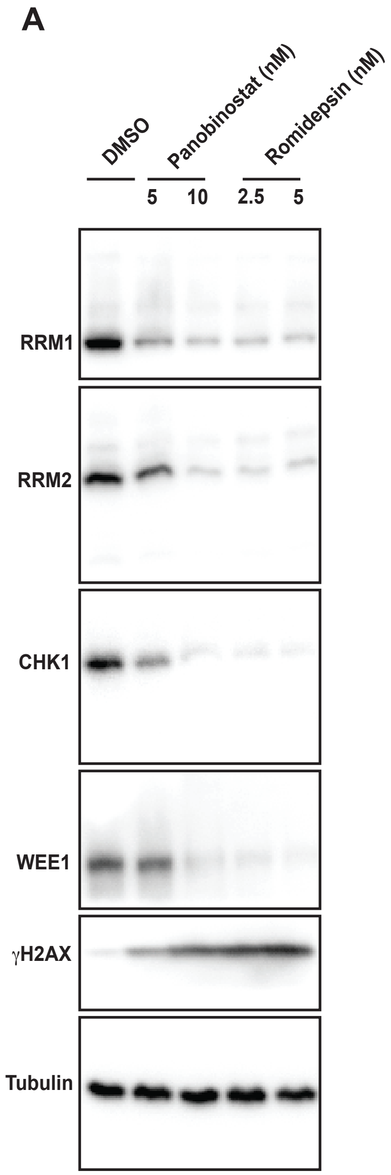

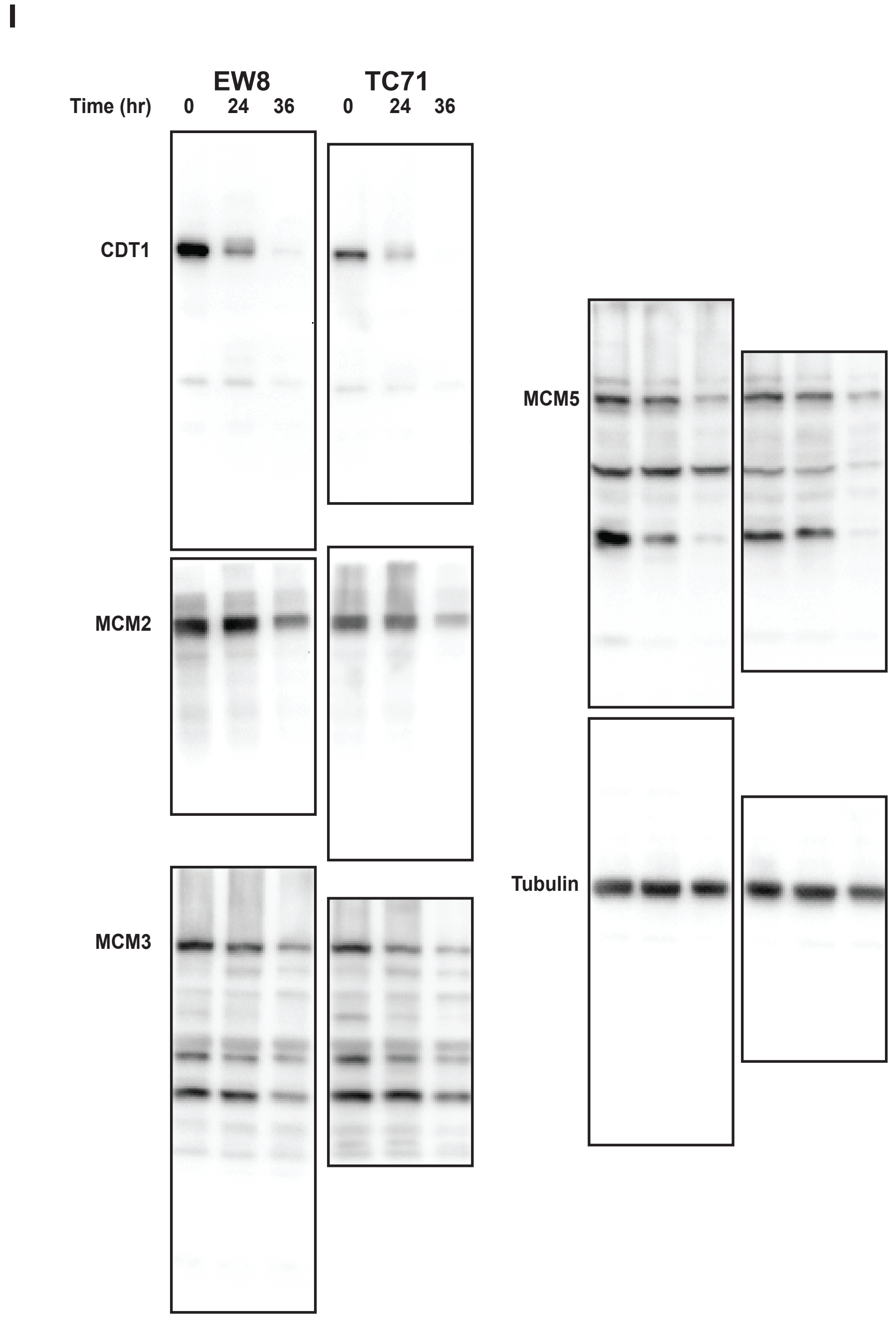

Full and unedited blots for Figure 3

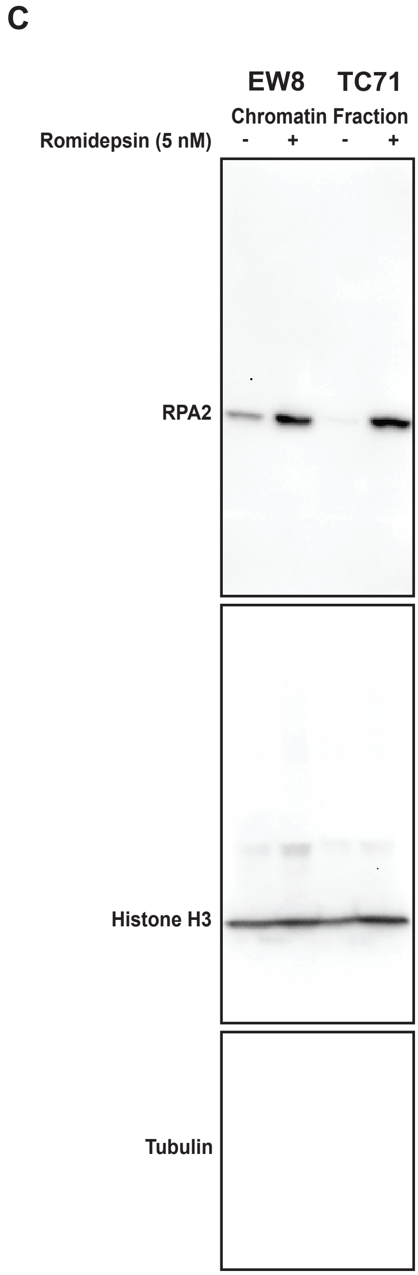

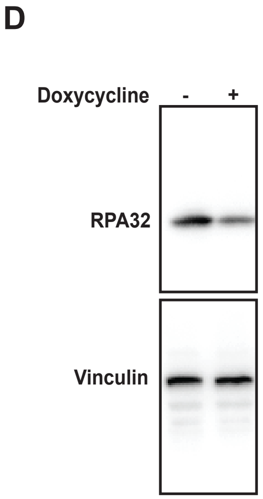

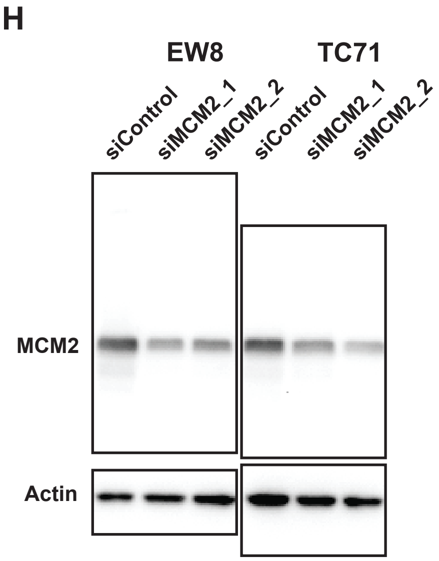

Full and unedited blots for Figure 4

F

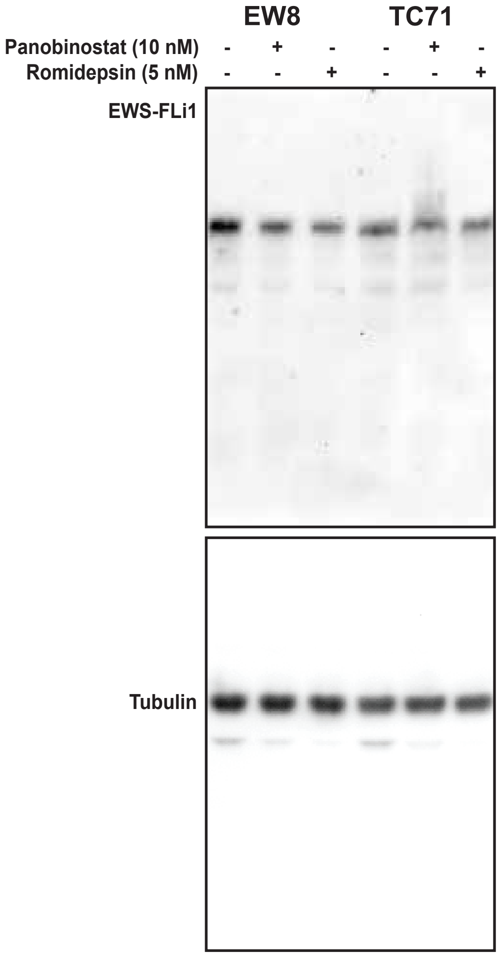

Full and unedited blots for Figure 5

E

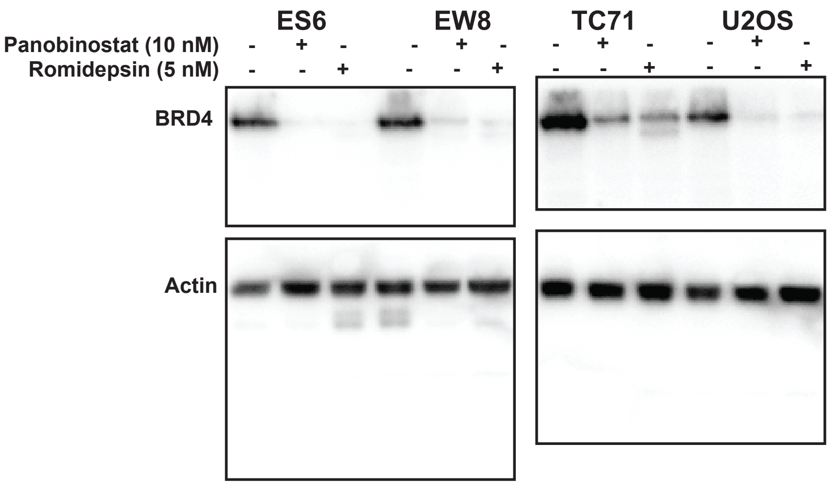

G

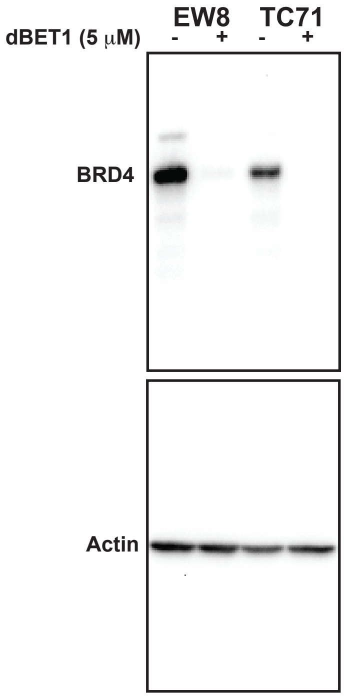

Full unedited blots for Figure 6

B

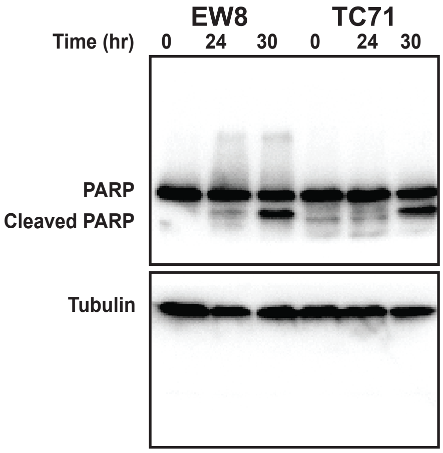

D

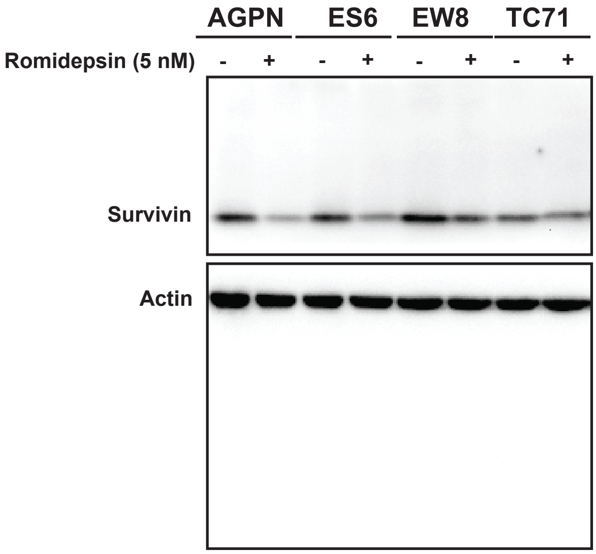

F

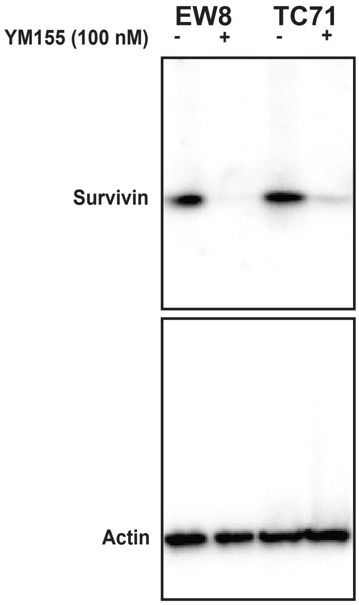

Full and unedited blots for Supplemental Figure 3

B

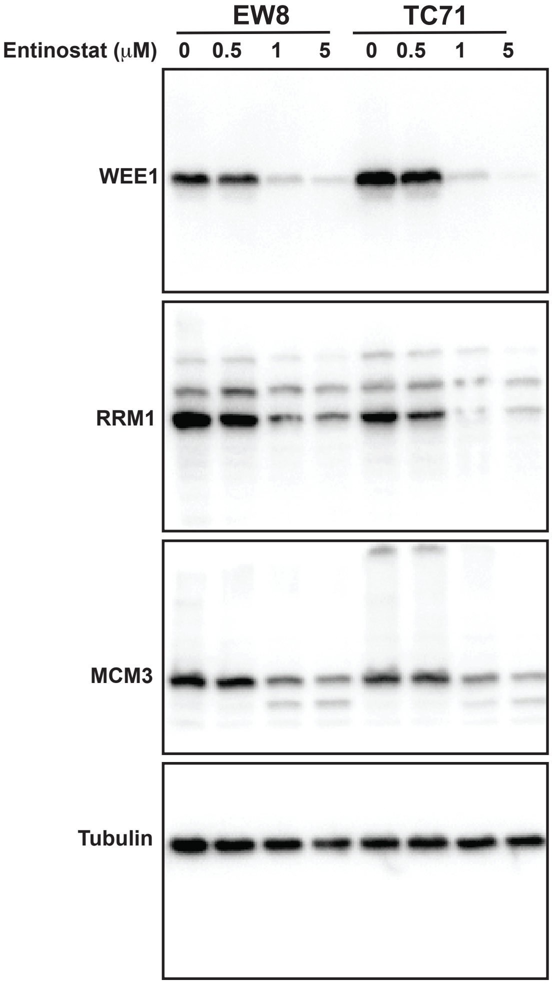

Full and unedited blots for Supplemental Figure 9

B

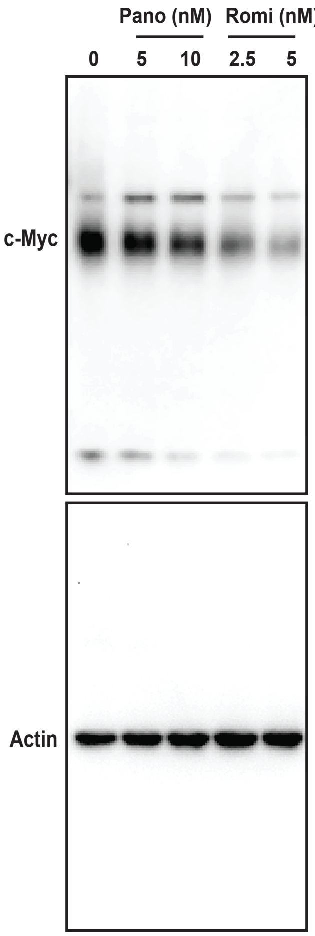

F

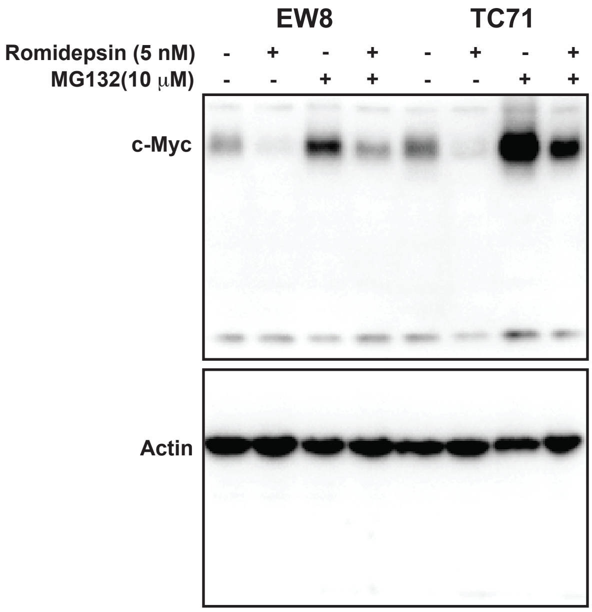

Supplement: Supplementary Data — Full and unedited blots [file crc-25-0058_supplementary_data_suppsd.pdf]
